# Dynamic networks of cortico-muscular interactions in sleep and neurodegenerative disorders

**DOI:** 10.3389/fnetp.2023.1168677

**Published:** 2023-09-05

**Authors:** Rossella Rizzo, Jilin W. J. L. Wang, Anna DePold Hohler, James W. Holsapple, Okeanis E. Vaou, Plamen Ch. Ivanov

**Affiliations:** ^1^ Keck Laboratory for Network Physiology, Department of Physics, Boston University, Boston, MA, United States; ^2^ Department of Engineering, University of Palermo, Palermo, Italy; ^3^ Department of Neurology, Steward St. Elizabeth’s Medical Center, Boston, MA, United States; ^4^ Department of Neurology, Boston University School of Medicine, Boston, MA, United States; ^5^ Department of Neurosurgery, Boston University School of Medicine, Boston, MA, United States; ^6^ Harvard Medical School and Division of Sleep Medicine, Brigham and Women Hospital, Boston, MA, United States; ^7^ Institute of Biophysics and Biomedical Engineering, Bulgarian Academy of Sciences, Sofia, Bulgaria

**Keywords:** network physiology, dynamic networks, time delay stability, brain waves, muscle tone, Parkinson’s, sleep, REM behavior disorder

## Abstract

The brain plays central role in regulating physiological systems, including the skeleto-muscular and locomotor system. Studies of cortico-muscular coordination have primarily focused on associations between movement tasks and dynamics of specific brain waves. However, the brain-muscle functional networks of synchronous coordination among brain waves and muscle activity rhythms that underlie locomotor control remain unknown. Here we address the following fundamental questions: what are the structure and dynamics of cortico-muscular networks; whether specific brain waves are main network mediators in locomotor control; how the hierarchical network organization relates to distinct physiological states under autonomic regulation such as wake, sleep, sleep stages; and how network dynamics are altered with neurodegenerative disorders. We study the interactions between all physiologically relevant brain waves across cortical locations with distinct rhythms in leg and chin muscle activity in healthy and Parkinson’s disease (PD) subjects. Utilizing Network Physiology framework and time delay stability approach, we find that 1) each physiological state is characterized by a unique network of cortico-muscular interactions with specific hierarchical organization and profile of links strength; 2) particular brain waves play role as main mediators in cortico-muscular interactions during each state; 3) PD leads to muscle-specific breakdown of cortico-muscular networks, altering the sleep-stage stratification pattern in network connectivity and links strength. In healthy subjects cortico-muscular networks exhibit a pronounced stratification with stronger links during wake and light sleep, and weaker links during REM and deep sleep. In contrast, network interactions reorganize in PD with decline in connectivity and links strength during wake and non-REM sleep, and increase during REM, leading to markedly different stratification with gradual decline in network links strength from wake to REM, light and deep sleep. Further, we find that wake and sleep stages are characterized by specific links strength profiles, which are altered with PD, indicating disruption in the synchronous activity and network communication among brain waves and muscle rhythms. Our findings demonstrate the presence of previously unrecognized functional networks and basic principles of brain control of locomotion, with potential clinical implications for novel network-based biomarkers for early detection of Parkinson’s and neurodegenerative disorders, movement, and sleep disorders.

## 1 Introduction

The human organism consists of diverse physiological systems with different structure, dynamic mechanisms of regulation, and particular functions necessary to maintain health (Bashan et al., 2012; Ivanov and Bartsch, 2014; Lehnertz et al., 2021; Schmal et al., 2022). The physiological cycles of human behaviors and activities during the day followed by restoring functions during night sleep require coordination and synchrony among systems that is facilitated through an integrated network of physiologic interactions (Healy et al., 2021; Ivanov, 2021; Mutti et al., 2022; Seok et al., 2022). Coordinated interactions between organ systems occur at multiple spatio-temporal scales, and a dysfunction or failure of one system can trigger a breakdown of the entire network, leading to collapse of the entire organism (Sinha et al., 2022; Tufa et al., 2022). Thus, to understand basic physiologic states and functions, and to fully characterize health and disease it is essential to identify and quantify the network of interactions among physiological systems. A new field, Network Physiology (Ivanov et al., 2016; 2017; Ivanov, 2021), has emerged with novel theoretical framework and methods tailored to investigate interactions among diverse systems across levels and time scales, leading to the discovery of dynamic networks underlying brain-organ and organ-organ interactions in relation to basic physiological states (Bashan et al., 2012; Faes et al., 2014; Bartsch et al., 2015; Liu K. K. L. et al., 2015; Faes et al., 2015; Porta and Faes, 2015; Lin et al., 2016; 2020), various conditions such as age, cognitive tasks/stress, rest and exercise, training and fatigue (Bartsch et al., 2012; Armstrong et al., 2022; Chen et al., 2022; Garcia-Retortillo and Ivanov, 2022; Papadakis et al., 2022) and pathological disorders (Moorman et al., 2016; Ivanov et al., 2021; Berner et al., 2022; González et al., 2022).

In the context of Network Physiology, the structure and dynamics of the regulatory networks underlying brain control of the locomotor system remain not understood. Uncovering the complexity of cortico-muscular interactions is challenging due to the large variety of muscles within the muscular system, where each muscle comprises muscle fibers of different type (fast and slow) with distinct speed of shortening in response to activation (Scott et al., 2001). Further, motor impulses originating in the motor area of the precentral gyrus in the cerebral cortex are transmitted to muscles by motor neurons with different frequency of activation, and distinct functional sets of locomotor modules within each muscle control locomotion (Leocani et al., 1997; Yokoyama et al., 2016; Rendeiro and Rhodes, 2018; Zandvoort et al., 2019). General associations between movements and cortical rhythms have shown changes in the spectral power of specific brain waves during particular tasks (Jasper and Penfield, 1949; Chatrian et al., 1958; Pfurtscheller and Aranibar, 1977; Leocani et al., 1997; Pfurtscheller and da Silva, 1999; Cheyne, 2013; Ciria et al., 2019). Synchronization of motor cortex dynamics and muscular activity has been established, employing cortico-muscular coherence (CMC) on specific frequency bands during muscular contraction (Conway et al., 1995; Brown et al., 1998; Liv Hansen and Bo Nielsen, 2004; Omlor et al., 2007; Boonstra et al., 2009; Cheyne, 2013). However, we do not know i) how brain rhythms interact as a network to control diverse muscle groups and motor units within muscles with different histochemical characteristics, and ii) how distinct frequency components reflecting muscle tone activation respond to bursts in brain rhythms to maintain body stability, movement and control.

Understanding brain control on the locomotor system and underlying dynamic networks of cortico-muscular interactions is essential for developing diagnostic approaches and treatment strategies of movement disorders (Harley, 1874; Carleton, 1931; Niemeyer, 1945; Cooper and Bravo, 1958; Tabaddor et al., 1978; Kronholm et al., 1993; Cohen-Solal et al., 1998), and neurodegenerative diseases such as Parkinson’s (PD) (Wolf et al., 1939; Lillie and Lake, 1949; Wiley, 1968; Nussbaum et al., 1992; Hurwitz et al., 1995; Brouillet et al., 1999; Popoli et al., 2002; Childs, 2004; Tuboly and Vécsei, 2013). Despite the vast literature of basic and clinical research on PD, there are no established markers of the disease onset, and it is hypothesized that PD starts years before appearance of evident symptoms related to locomotor dysfunction, such as resting tremors, slow movement, rigid muscles, unsteady gait and freezing of gait. Non-motor symptoms, related to autonomic nervous system dysfunction with effects on blood flow fluctuations, sleep, mood, cognition, sense of smell, constitute the so-called Prodromal-PD, referring to the stage at which individuals do not fulfill diagnostic criteria for PD (i.e., bradykinesia and at least one other motor sign) but do exhibit signs and symptoms that indicate a higher than average risk of developing motor symptoms and a diagnosis of PD in the future (Postuma et al., 2012; Postuma and Berg, 2019). Recent works have found correlations between disease severity and elevated spectral power of the *β*-cortical rhythm (Benabid, 2003), as well as synchronization of *θ* and *β* rhythms across cortical areas (Stoffers et al., 2008; Krause et al., 2014), phase amplitude coupling (PAC) between *β*-phase (13 − 30 Hz) and *γ*-amplitude (50 − 200 Hz) in local field potentials (De Hemptinne et al., 2013), EEG-EEG coherence in the 10 − 35 Hz range (Silberstein et al., 2005), and EEG-EMG coherence in the 5 − 18 Hz range (Caviness et al., 2006). Treatments such as Deep Brain Stimulation (DBS) were found to reduce EEG-EEG coherence, cortical EEG PAC (Silberstein et al., 2005; Weiss et al., 2015; Oswal et al., 2016), and EEG-EMG coherence (Timmermann et al., 2004; De Hemptinne et al., 2015), correlated with clinical improvement, as does L-DOPA and dopaminergic therapy (Swann et al., 2015; Madadi Asl et al., 2022). Further, cortical hyperconnectivity, increased oscillatory neural activity of the basal ganglia and heightened synchronous activity across the basal ganglia thalamocortical networks have been observed in PD patients off medication, while administration of L-DOPA has been reported to downregulate this hyperconnectivity (Costa et al., 2006; Gatev et al., 2006; Hammond et al., 2007; Eusebio and Brown, 2009). Amplified PAC between *β* rhythm and high-frequency oscillations (200 − 500 Hz) was also observed at the dorsal border of subthalamic nucleus, closest to the contact used for DBS (Özkurt et al., 2011; Yang et al., 2014) and is significantly reduced with dopaminergic medication (López-Azcárate et al., 2010; Hirschmann et al., 2013; van Wijk et al., 2016). While these empirical findings have focused on coordinated activity of specific cortical rhythms and on cortico-muscular coherence within limited frequency bands to probe cortico-muscular regulation, quantify effects of PD, and demonstrate that brain stimulation with impulses at a certain frequency reduces PD symptoms (Benabid, 2003; Bronstein et al., 2011), it remains unknown whether all brain waves across cortical locations play role in muscle activity control, and how different brain waves synchronize as a network with distinct rhythms embedded in muscle activity. Here we hypothesize that mapping cortico-muscular interactions among all brain waves and muscle activity rhythms in different muscles can provide a comprehensive picture of locomotor control, and lead to novel network-based markers of autonomic nervous system dysfunction in PD for early diagnosis.

Interactions between physiological systems under neuro-autonomic regulation, including distinct forms of cardio-respiratory coupling, change in response to modulation of autonomic function across the sleep-wake cycle and different sleep stages (Bartsch et al., 2012; Bartsch et al., 2014; Bartsch and Ivanov, 2014; Borovkova et al., 2022). Thus, we hypothesize that cortico-muscular interactions and the underlying dynamic networks change with transitions across physiological states where autonomic control is dominant. To facilitate movements the locomotor system requires continuous coordination of various muscle groups, synchronous activity of motor neurons and coordination among neuronal population in the motor cortex and other brain areas (Kerkman et al., 2018; Boonstra et al., 2019; Garcia-Retortillo et al., 2020; Garcia-Retortillo and Ivanov, 2022; Kerkman et al., 2022; Rizzo et al., 2022). Correspondingly, investigations on brain control of locomotion in both healthy and PD subjects have focused on correlated dynamics among cortical rhythms across cortical areas (e.g., motor cortex, hippocampus) in response to a given movement (Silberstein et al., 2005; Wheaton et al., 2005; Stoffers et al., 2008; De Hemptinne et al., 2013), or on the coherence between a given cortical rhythm with peripheral muscle activity during walking, targeted movement tasks or exercise (Caviness et al., 2006; Muthuraman et al., 2012; Krause et al., 2014; Kato et al., 2016; Scholten et al., 2016; Zehr et al., 2016; Rendeiro and Rhodes, 2018; Günther et al., 2019). However, default locomotor control even at rest, without specific tasks and targeted movements, requires coordination and synchronous activation of muscle fibers, muscles and muscle groups, as well as integration of cortico-muscular interactions. Yet, the functional networks underlying the communication between all cortical rhythms across cortical areas and all rhythms embedded in default muscular activity during rest and physiological states where autonomic control is dominant (e.g., sleep, sleep stages) have not been investigated.

Autonomic sleep regulation is affected in the early stage of PD, prior to the onset of motor symptoms that are traditionally used for diagnosis. PD patients frequently exhibit difficulty maintaining sleep as well as fragmented sleep with frequent awakenings (Chaudhuri et al., 2002; McKeith et al., 2005; Cochen De Cock and Arnulf, 2008). Sleep architecture is significantly altered with PD, characterized by reduced rapid-eye-movement (REM) and deep sleep (DS), and pronounced REM sleep behavior disorder (RBD, i.e., a parasomnia in REM associated with dream enacting behaviors, resulting from absence of normal muscle atonia during REM sleep stage) (Gagnon et al., 2002; Iranzo et al., 2006; Postuma et al., 2009). RBD may precede traditional locomotor-symptoms based PD diagnosis by an average of 12 years, and could serve as a early biomarker. While research on brain control of locomotion in PD has mainly focused on EEG-EMG coupling and same frequency coherence patterns during movement (Chen et al., 2012; 2013b; a,c; Roeder et al., 2020), only a few studies have investigated PD alteration of autonomic function during sleep reporting change in connectivity between different cortical rhythms (Higginbotham et al., 2019). Despite the potential for early diagnosis, there are no studies on the complex integration network of interactions between all brain waves and rhythms of muscle activity under autonomic regulation during sleep, and how changes in autonomic control with PD during sleep alters these cortico-muscular networks. In the Network Physiology framework of building an atlas of network maps that represent physiologic interactions between diverse organ systems, the presented here approach aims to reconstruct and analyze in detail the functional networks representing cortico-muscular dynamics under autonomic control across sleep stages. Further, we established novel network-based makers of autonomic regulation of cortico-muscular interactions that could lead to early diagnosis of PD, guide and assess effects of medications and outcomes of treatment strategies.

It has been recently found that pairs of brain waves interact through distinct coupling functions, and that physiological states (sleep/wake, sleep stages, rest/exercise, cognitive tasks) and conditions (age maturation) are uniquely characterized by an ensemble of coupling forms among brain waves and by specific network topology necessary to facilitate physiological functions (Liu K. K. et al., 2015; Lin et al., 2020; Chen et al., 2022; Hu et al., 2022; Martinez-Gutierrez et al., 2022; Ramos-Loyo et al., 2022). Such functional networks among brain waves are expression of synchronization mechanisms integrating different neuronal networks (Wu et al., 2022). Moreover, it was discovered that not only brain-brain but also brain-muscle interactions reflect changes in physiologic regulation, where physiologic states are associated with specific networks of dynamic interactions of all brain rhythms with the rhythms embedded in muscle activity. (Rizzo et al., 2020; 2022). Further, the established composition of slow and fast muscle fibers in different muscles for generating specific functions (Garcia-Retortillo et al., 2020; 2021), and the presence of dynamic networks underlying synchronous muscle fibers activation and interactions among muscles during different states (rest/exercise) and conditions (fatigue) (Balagué et al., 2020; Garcia-Retortillo and Ivanov, 2022), motivate our investigation of the role that different rhythms (frequency domains) of muscle activation play in cortico-muscular networks across physiologic states under healthy condition and how these networks are altered with neurodegenerative disorders such as Parkinson’s. Here we study how all physiologically-relevant brain rhythms across cortical locations synchronize their bursting activity with rhythms in the peripheral muscle tone during overnight sleep for four basic physiological states–quiet Wake, REM, Light Sleep (LS), Deep Sleep (DS). We analyze continuous and synchronously recorded EEG and EMG signals during night-time sleep to derive functional brain-muscle interaction networks, when specific tasks and targeted movements are not present. In the absence of conscious movements, changes in brain-muscle network dynamics with transitions across resting states and sleep stages reflect solely changes in autonomic regulation.

To derive network maps representing synchronization and functional pathways of cross-communication between brain dynamics and peripheral muscle activity, and to uncover basic principles of locomotor control from the hierarchical organization of these networks, we use Network Physiology framework (Ivanov and Bartsch, 2014; Ivanov et al., 2016; 2017) and a method based on the concept of Time Delay Stability (TDS) (Bashan et al., 2012) (see Methods 2.3). We establish first detailed functional networks and brain-muscle interaction frequency profiles that uniquely define resting wake and sleep stages. We establish how cortico-muscular networks evolve and reorganize with transitions across physiologic states, comparing age-matched populations of healthy and Parkison’s subjects. While effects of Parkinson’s neurodegeneration are traditionally studied during walking or targeted movements, the reported here findings provide a new picture that demonstrates i) active network coordination among cortical and muscle rhythms even at a sub-activity level, and that ii) cortico-muscular networks are dramatically altered with PD even during sleep—i.e., breakdown of network interactions during wake, LS and DS, and overexpression of interactions during REM. Our findings demonstrate that even in the absence of conscious targeted movements, neurodegenration due to PD dramatically changes muscle regulation across different physiological states under dominant autonomic regulation. Further, the uncovered dynamic patterns in cortico-muscular networks and their alteration with PD provide essentially new information about the nature of REM behavior disorder, and demonstrate the utility of our approach to derive novel network-based biomarkers of early PD diagnosis based on the effects of autonomic regulation dysfunction on cortico-muscular networks.

## 2 Methods

### 2.1 Data

We analyze continuous synchronously recorded surface EEG and EMG signals during night-time sleep from 97 healthy subjects (51 female, 46 male, age average 67.4 ± 10.5 years) and 33 age-matched Parkinson’s disease (PD) subjects (9 female, 24 male, age average 71.0 ± 10.3 years). Polysomnographic (PSG) recordings have average duration of 8.0 h for healthy subjects and 7.1 h for PD subjects.

Subjects in the PD group have the following stratification by disease severity Hoehn *&* Yahr stage: 5 subjects with stage 1–1.5; 16 subjects with stage 2–2.5; 8 subjects with stage 3; 3 subjects with stage 4, and 1 subject with stage 5. Hoehn *&* Yahr stage 1 represents the earliest stage with usually minimal (or no) functional impairment, and stage 5 is the most advanced stage, characterized by wheelchair or bed confinement. PD patients are on different medications depending on their specific conditions. Medications that would be effective during nighttime sleep are Carbidopa/Levodopa CR, Dopamine agonists and MAOB inhibitors. Information regarding the time of last dose medication intake prior to PSG testing and sleep onset was not recorded. Subjects in the PD group take their last dose before bedtime and do not take any medications during the night.

Based on the American Association of Sleep Medicine (AASM) guidelines, standard sleep lab protocols and PSG recordings include six EEG channels (two frontal, two central and two occipital for the left- and right-brain hemisphere), and surface EMG channels from the anterior tibialis (leg) muscle and the mentalis (chin) muscle. For both healthy and Parkinson’s group in our study the EMG channel was derived from the left leg muscle (standard PSG recordings do not include leg EMG channels from both limbs). PSG data are divided in 30 s epochs and scored as Wake, REM, Light Sleep (LS) and Deep Sleep (DS). Sleep stage scoring was performed by certified sleep lab technicians based on standard criteria (Rechtschaffen and Kales, 1968; Klösch et al., 2001). In the PD group 7 subjects never reached REM sleep during over night sleep, 1 subject did not have LS, and 12 PD subjects and 3 healthy subjects did not reach DS—thus, these subjects are not taken into account in our analysis of the corresponding sleep stages.

Analyzed data include EEG signals (sampling rate 200 Hz for 55 healthy subjects and for all 33 PD subjects; 256 Hz for 42 healthy subjects) from six scalp locations—frontal left: Fp1 for healthy subjects and F3 for PD subjects; frontal right: Fp2 for healthy subjects and F4 for PD subjects; central left C3, central right C4, occipital left O1, and occipital right O2 for both healthy and PD groups. EEG reference electrodes are M1 for the right brain hemisphere and M2 for the left brain hemisphere placed on the left and right mastoids respectively. EMG signals are derived from chin and left leg muscle (sampling rate 200 Hz for 55 healthy subjects and for all 33 PD subjects, 256 Hz for 42 healthy subjects). Before mounting EMG electrodes, participants’ skin is shaved and cleaned using alcohol and left to dry for 60 s to reduce myoelectrical impedance. EMG electrode for the anterior tibialis left leg muscle (pre-gelled Ag/AgCl bipolar surface electrodes) are placed at 1/3 of the line between the tip of the fibula and the tip of the medial malleolus, and their orientation corresponds to the direction of the line between the tip of the fibula and the tip of the malleolus. For the healthy dataset the reference electrode for the anterior tibialis is located on the ankle, and the interelectrode distance is 2 cm. For the mentalis (chin muscle), 8-mm-diameter surface pre-gelled electrodes are placed on the mentalis equidistant to the median line with an inter-electrode distance of 1 cm, and the ear lobe is used as a reference point. For the PD database, chin and leg EMGs are considered bipolar leads, i.e., the outputs come from 2 leads placed in the same area, roughly 2–3 cm apart. After the electrodes are secured, a quality check is performed to ensure EMG signal validity. Healthy subjects data in this study are multi-channel physiologic recordings from the EU SIESTA database (Klösch et al., 2001). PD subjects data are collected at the Sleep Disorder Center, Boston Medical Center.

We focus on network dynamics during sleep as sleep stages are well-defined physiological states under neuroautonomic regulation, when physical activity, external influences and sensory inputs are greatly reduced (Goodfellow et al., 2022). Thus, the data structure allows i) to investigate the dynamics and organization of functional networks representing cortico-muscular interactions during distinct physiological states (sleep stages) with different autonomic control; ii) to study how these networks reorganize and breakdown with neurodegeneration due to Parkinson’s, with the aim to uncover universal network characteristics and basic laws of brain-muscle control; iii) derive novel biomarkers for early diagnosis based on PD effect on autonomic regulation.

### 2.2 Signal preprocessing

To compare EEG and EMG signals and study their physiological interaction the spectral power of seven EEG and EMG frequency bands was extracted in moving windows of 2 s with a 1 s overlap: *δ* (0.5 − 3.5 Hz), *θ* (4 − 7.5 Hz), *α* (8 − 11.5 Hz), *σ* (12 − 15.5 Hz), *β* (16 − 19.5 Hz), *γ*_1_ (20 − 33.5 Hz) and *γ*_2_ (34 − 100 Hz). This defines a time series *S* ^*ν*^–with *ν* = 1, … , *N*, and *N* number of windows–for each frequency band, with a temporal resolution of 1 s. The spectral power *S*(*f*) has been calculated as *S*(*f*) = |*F*(*f*)|^2^/(*W* ⋅ *F*_*s*_), where *F*(*f*) is the Fourier transform, *W* is the window size, and *F*_*s*_ is the sampling frequency (Rizzo et al., 2020). The Fourier transform has been evaluated using the fast Fourier transform (FFT) algorithm in Matlab. The spectral power in a given window *ν* and in a given frequency band Δ*f* is defined as

$$S^{\nu }\left(\Delta f\right)=\int_{f_{1}}^{f_{2}}S^{\nu }\left(f\right)df$$

where *f*_1_ and *f*_2_ are the lower and upper bound of the band.

### 2.3 Time delay stability (TDS) method

The time delay stability (TDS) method is a novel approach specifically developed to identify and quantify pair-wise coupling and network interactions of diverse dynamical systems (Bashan et al., 2012). The idea that led to the development of the TDS method to analyze the interactions between organs under neural regulation was the observation of synchronous bursts in the signals coming from different organ systems, underlying a general event occurring in all systems and a transition from one physiological state to another.

**FIGURE 1:**
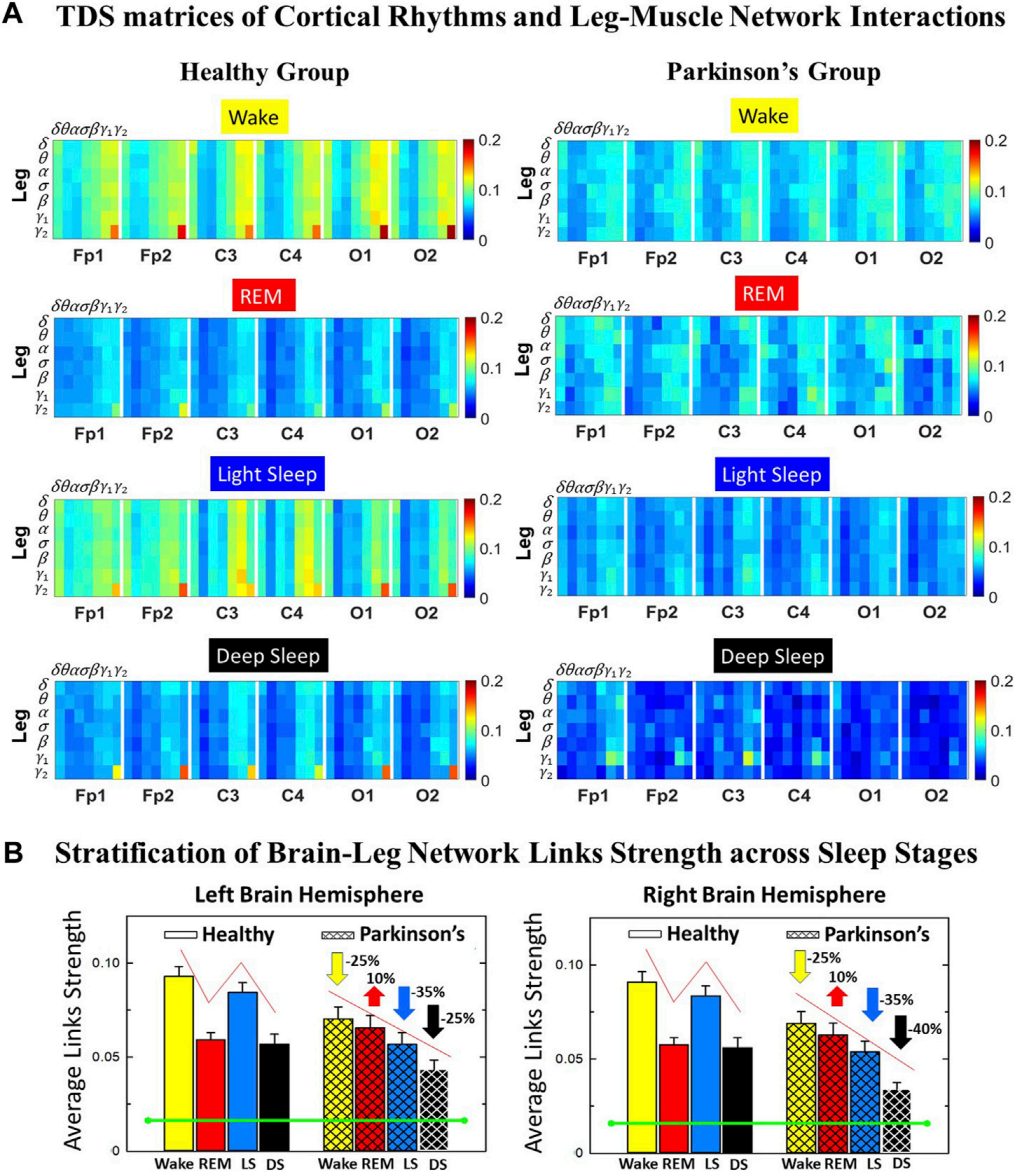
Time Delay Stability (TDS) matrices representing brain-leg network interactions across physiologic states in healthy and Parkinson’s subjects. **(A)** Group-averaged TDS matrices represent physiological interactions between brain and leg-muscle tone during Wake, REM, light and deep sleep in healthy (left panels) and PD subjects (right panels). Matrix elements show coupling strength between seven physiologically-relevant cortical rhythms (*δ*, *θ*, *α*, *σ*, *β*, *γ*_1_, *γ*_2_) derived from six EEG channels (*x*-axis: Frontal Fp1 and Fp2; Central C3 and C4; Occipital O1 and O2) and the corresponding EMG frequency bands (*y*-axis) representing leg muscle activation. Coupling (network links) strength is quantified by the fraction of time (out of the total duration of a given sleep stage throughout the night) when TDS is observed. Matrix elements are obtained by quantifying the TDS for each pair of EEG vs. EMG bands after calculating the weighted average across all subjects in each group (Methods Section 2.3). Color code indicates TDS coupling strength. **(B)** Histograms of links strength in the brain-leg network during different sleep stages for healthy and PD subjects for the left (left panel) and right (right panel) brain hemisphere. Group-averaged links strength is obtained using the TDS measure, where each bar represents the average strength of interactions of all cortical rhythms from all brain areas (Frontal, Central and Occipital) in each brain hemisphere with all leg muscle tone EMG bands. Error bars represent the standard error obtained for all subjects in each group; horizontal green lines in both panels mark a surrogate test threshold (*%TDS* =2.3%; Section Method 2.4) above which network interactions are physiologically significant. In healthy subjects brain-leg network interactions exhibit pronounced sleep-stage stratification: strong coupling during Wake and LS, and weaker during REM and DS. The Wake-REM-LS-DS alternation seen in healthy subjects is not present in PD, where a gradual decline is observed in links strength from Wake to DS. The change in the sleep-stage pattern is due to a moderate increase 10% for PD subjects in links strength during REM (*p* < 10^−3^ Wilcoxon test), and a dramatic decrease 25% − 40% during Wake and non-REM (NREM) sleep stages compared to healthy.

The TDS method is based on the concept of the time delay stability. Integrated physiologic systems are coupled by non-linear feedback and/or feed forward loops with a broad range of time delays. Thus bursting activities in one system are always followed by bursts in signals from other coupled systems. TDS quantifies the stability of the time delay with which bursts in the output dynamics of a given system are consistently followed by corresponding bursts in the signal output of other systems—periods with constant time delay between bursts in two systems indicate stable interactions. Correspondingly stronger coupling between systems results in longer periods of TDS (Figure 1–Figure 3). Thus, the links strength in the physiologic networks we investigate is determined by the percentage of the time when TDS is observed: higher percentage of TDS (%TDS) corresponds to stronger links.

**FIGURE 2:**
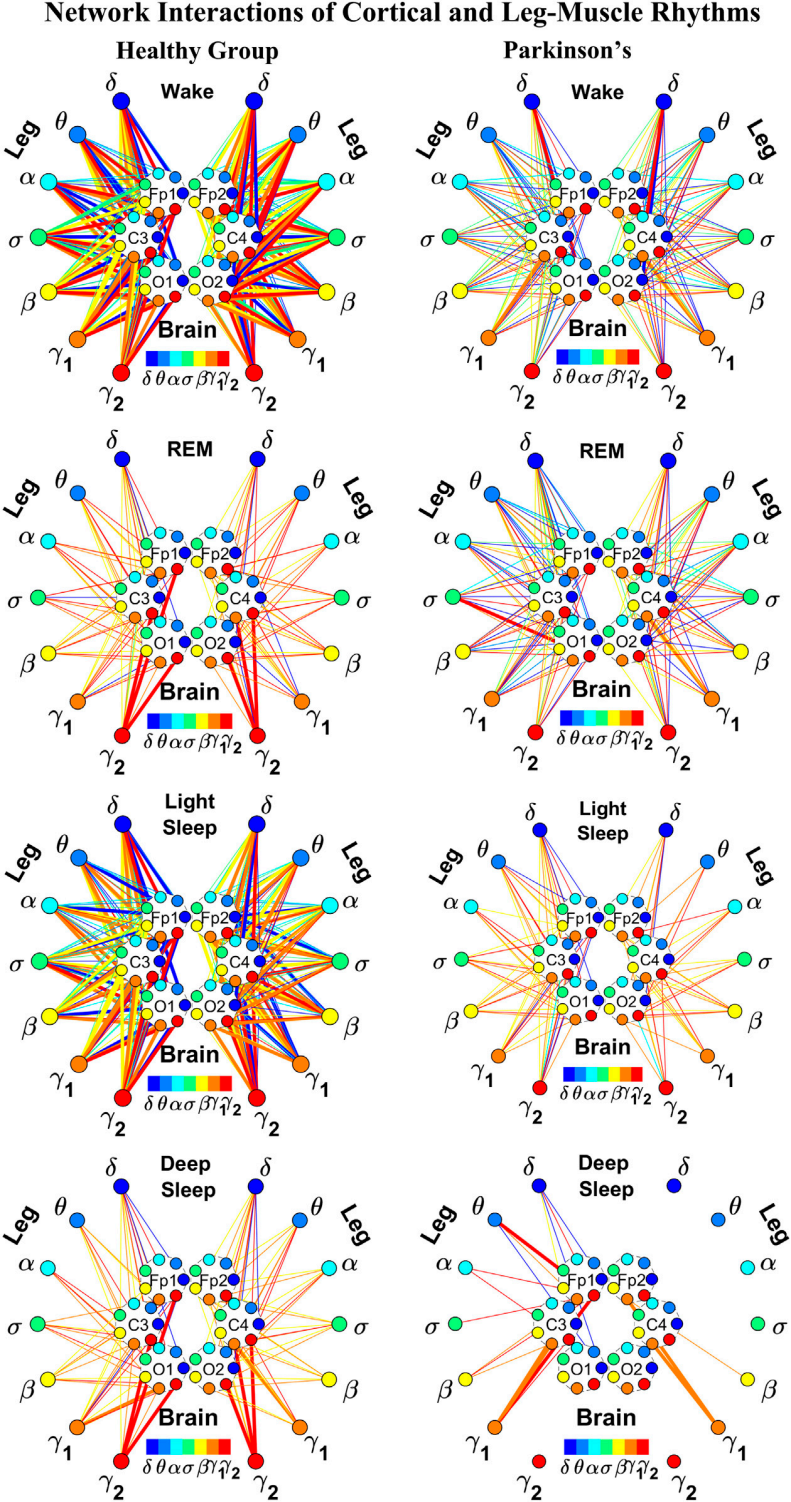
Reorganization in network topology of brain-leg interactions across physiological states and breakdown with Parkinson’s disease. Network maps are obtained based on the group-averaged TDS matrices in Figure 1 representing brain-leg interactions during Wake, REM, light and deep sleep for healthy (left networks) and PD subjects (right networks). Network links correspond to the TDS matrix elements, and show the coupling strength between seven physiologically relevant brain waves (*δ*, *θ*, *α*, *σ*, *β*, *γ*_1_, *γ*_2_) across cortical locations and leg muscle tone EMG frequency bands. Brain areas are represented by Frontal (Fp1 and Fp2), Central (C3 and C4) and Occipital (O1 and O2) EEG channels, where color nodes in each brain area represent distinct brain waves. Peripheral nodes indicate corresponding EMG frequency bands of leg muscle tone shown in same color code as the brain waves. Links reflect the coupling strength between cortical rhythms at different locations and EMG frequency bands as quantified by the TDS measure (Methods 2.3). Color of the links correspond to the cortical rhythm involved in that particular interaction. Links strength is marked by line width—thin lines for 6% < *%TDS* < 9% for Wake, REM, and LS, 6% < *%TDS* < 8% for DS; thick lines for *%TDS* > 9% for Wake, REM, and LS, *%TDS* > 8% for DS. Shown are all links above a threshold *%TDS* = 6%. The interaction networks in PD subjects during Wake, light and deep sleep are less dense compared to healthy, leading to a change in the sleep-stage stratification pattern.

**FIGURE 3:**
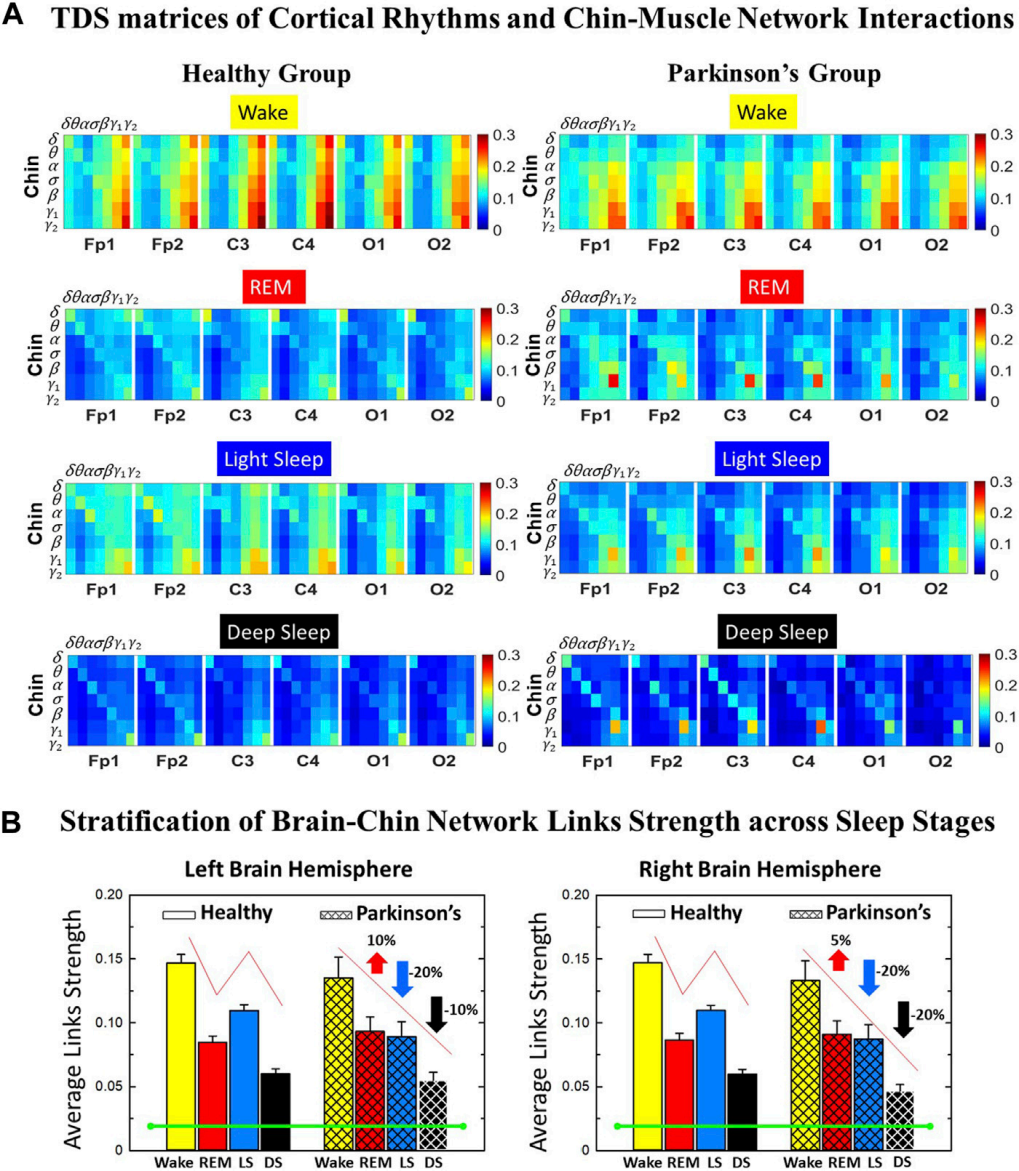
Time Delay Stability (TDS) matrices representing brain-chin network interactions across physiologic states in healthy and Parkinson’s subjects. **(A)** Group-averaged TDS matrices represent physiological interactions between brain and chin-muscle tone during Wake, REM, light and deep sleep in healthy (left panels) and PD subjects (right panels). Matrix elements show coupling strength between seven physiologically-relevant cortical rhythms (*δ*, *θ*, *α*, *σ*, *β*, *γ*_1_, *γ*_2_) derived from six EEG channels (*x*-axis: Frontal Fp1 and Fp2; Central C3 and C4; Occipital O1 and O2) and the corresponding EMG frequency bands (*y*-axis) representing chin muscle activation. Coupling (network links) strength is quantified by the fraction of time (out of the total duration of a given sleep stage throughout the night) when TDS is observed. Matrix elements are obtained by quantifying the TDS for each pair of EEG vs. EMG bands after calculating the weighted average across all subjects in each group (Methods Section 2.3). Color code indicates TDS coupling strength. **(B)** Histograms of links strength in the brain-chin network during different sleep stages for healthy and PD subjects for the left (left panel) and right (right panel) brain hemisphere. Group-averaged links strength is obtained using the TDS measure, where each bar represents the average strength of interactions of all cortical rhythms from all brain areas (Frontal, Central and Occipital) in each brain hemisphere with all chin muscle tone EMG bands. Error bars represent the standard error obtained for all subjects in each group; horizontal green lines in both panels mark a surrogate test threshold (*%TDS* = 2.3%; Section Method 2.4) above which network interactions are physiologically significant. Results for brain-chin interactions are similar to those found in brain-leg interactions (Figure 1). The network interactions in PD subjects exhibit a dramatic decrease 20% of coupling strength during LS and DS compared to controls (statistically significant difference – *p* ≤ 10^−2^ Wilcoxon test–during LS and borderline significant during DS), and a moderate increase 5% − 10% in links strength during REM (*p* ≤ 10^−2^ Wilcoxon test). This difference in PDs disrupts the W-REM-LS-DS pattern, observed in healthy individuals.

The TDS method (Bashan et al., 2012) to quantify the interaction between distinct physiologic systems A and B consists of the following steps. Consider the output signals {*a*} of system A and the output signal {*b*} of system B, each of length *N*. Divide both signals {*a*} and {*b*} into *N*_*L*_ overlapping segments *ν* of equal length *L* = 60*s*. Here we choose an overlap of *L*/2 = 30*s*, which corresponds to the time resolution of conventional sleep-stage-scoring epochs, and thus *N*_*L*_ = ⌊2*N*/*L*⌋ − 1, where ⌊2*N*/*L*⌋ is the largest integer *k* such that *k* ≤ 2*N*/*L*. Normalize the signals separately in each segment *ν* to zero mean and unit standard deviation in order to remove constant trends in the data and to obtain dimensionless signals. This normalization procedure assures that the estimate coupling between the signals {*a*} and {*b*} is not affected by their relative amplitudes. Then, calculate the cross-correlations

$$C_{ab}^{\nu }\left(\tau \right)=\frac{1}{L}\overset{L}{\underset{i=1}{\sum }}a_{i+\left(\nu -1\right)L\backslash 2}^{\nu }b_{i+\left(\nu -1\right)L\backslash 2+\tau }^{\nu }$$

between {*a*} and {*b*} in each segment *ν* using periodic boundary conditions. For each segment *ν*, estimate the time delay $\tau_{0}^{\nu }$ as the maximum in the absolute value of the cross-correlation function $C_{ab}^{\nu }\left(\tau \right)$ in the segment.

These steps result in a new temporal series of time delays $\left\{\tau_{0}^{\nu }{|}_{\nu \in \left\{1,\ldots ,N_{L}\right\}}\right\}$ describing the temporal evolution of the cross-talk between signals {*a*} and {*b*}. Time periods of stable interrelation between two signals are represented by segments of approximately constant *τ*_0_ in the series of time delays. In contrast, the absence of stable coupling between the signals corresponds to large fluctuations in *τ*_0_. To identify periods of stable coupling, the series of time delays is scanned using a 5 points sliding window (corresponding to a window of 5 × 30*s* consecutive segments *ν*) with step size 1. Periods are labelled as stable when at least four out of five points the time delay remains in the interval [*τ*_0_ − 1, *τ*_0_ + 1]. The %TDS is finally calculated as the fraction of stable points in the time series $\left\{\tau_{0}^{\nu }\right\}$ , and is a measure of the coupling strength between two systems A and B. Periods with stable time delay are characterized by constant *τ*_0_. Long periods of constant time delay *τ*_0_ indicate strong TDS coupling (Rizzo et al., 2020). The novel concept of Time Delay Stability defines a new metric for the link strength between two different systems.

### 2.4 Surrogate tests and significance threshold for network links strength

To test the statistical significance and physiological relevance of the network interactions identified by the TDS method, we perform a surrogate test to establish a threshold of significance for links strength (Rizzo et al., 2020). Statistical significance is estimated by comparing the strength distribution of a given link obtained from all subjects in a given sleep stage with the distribution of the corresponding surrogate link representing ‘interactions’ between the same two systems paired from different subjects.

A significance threshold for network links strength is determined performing the following steps: for each link in a given sleep stage, 200 surrogates are generated considering signals from two distinct and randomly chosen subjects, and a surrogate average link strength (%TDS) is obtained. The procedure is repeated for each network link to obtain a distribution of surrogate link strengths in each sleep stage. For each distribution the mean *μ*_*surr*_ and standard deviation *σ*_*surr*_ are estimated. Thus, the significance threshold at 95% confidence level for the network links strength is defined as *μ*_*surr*_ + 2*σ*_*surr*_ for each sleep stage (Rizzo et al., 2020). The significance threshold is represented by horizontal green lines in all figure panels showing bar plots of average links strength.

### 2.5 Cortico-muscular interaction networks

#### 2.5.1 TDS matrix and network link definition

The TDS matrix consists of the pairwise coupling strength between seven cortical rhythms (*δ*, *θ*, *α*, *σ*, *β*, *γ*_1_ and *γ*_2_) derived from an EEG channel and EMG frequency bands representing leg (Figure 1A) and chin muscle activation (Figure 3A). The coupling strength between two signals is defined as the percentage of time over which TDS is observed, i.e., $\%TDS=\left(\sum_{i}^{N_{L}}s_{i}\right)/L\cdot 100$ where *s*_*i*_ is 1 if the corresponding *i*th segment is labelled as stable for the TDS measure or 0 if the corresponding *i*th segment is labelled as unstable for the TDS measure and *L* is the total duration of signals (Rizzo et al., 2020).

For each physiologic state, we calculate a group-average TDS matrix for couplings of all leg EMG (chin EMG) frequency bands with each cortical rhythm from each of the EEG channels (Frontal Fp1 and Fp2 for healthy, and F3 and F4 for PD subjects, Central C3 and C4, Occipital O1 and O2 for both groups of subjects). In these matrices each element represents the TDS coupling strength between signal *a* and *b* during a given sleep stage *s* averaged over all subjects for each group (healthy in left panels of Figure 1A and Figure 3A, and PD in right panels of Figure 1A and Figure 3A) and defined as:

$${\bar{\%TDS}}_{ab}^{s}=\frac{\sum_{i=1}^{M}TDS_{i}\cdot L_{i}^{s}}{\sum_{i=1}^{M}L_{i}^{s}}\cdot 100,$$ (1)

where $L_{i}^{s}$ represents the total duration of a given sleep stage *s* for subject *i*, and *TDS*_*i*_ stands for the TDS coupling strength between signal *a* and *b* for sleep stage *s* obtained from subject *i*.

##### 2.5.1.1 Graphical visualization of the full cortico-muscular network

**FIGURE 4:**
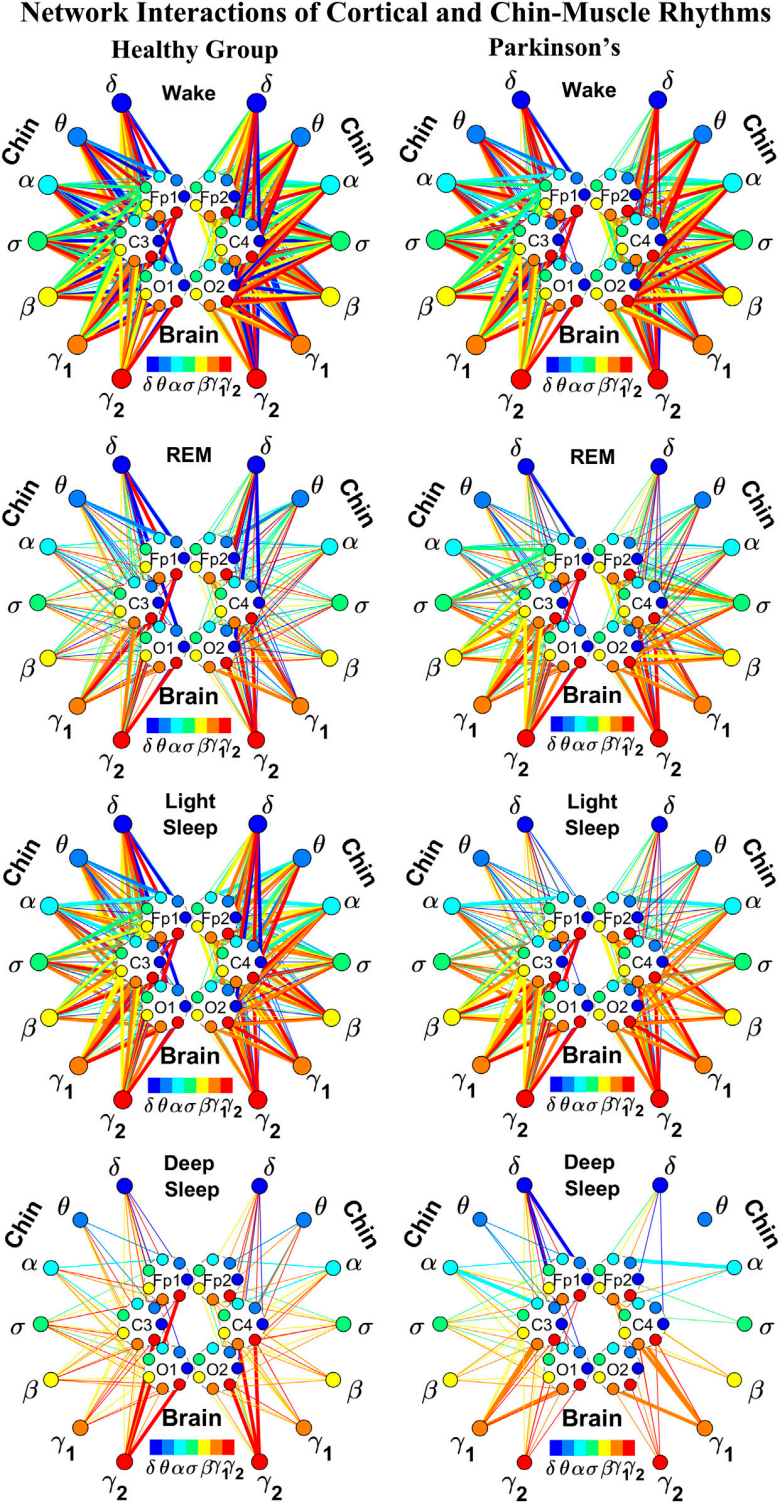
Reorganization in network topology of brain-chin interactions across physiological states and breakdown with Parkinson’s disease. Network maps are obtained based on the group-averaged TDS matrices in Figure 3 representing brain-chin interactions during Wake, REM, light and deep sleep for healthy (left networks) and PD subjects (right networks). Network links correspond to the TDS matrix elements, and show the coupling strength between seven physiologically relevant brain waves (*δ*, *θ*, *α*, *σ*, *β*, *γ*_1_, *γ*_2_) across cortical locations and chin muscle tone EMG frequency bands. Brain areas are represented by Frontal (Fp1 and Fp2), Central (C3 and C4) and Occipital (O1 and O2) EEG channels, where color nodes in each brain area represent distinct brain waves. Peripheral nodes indicate corresponding EMG frequency bands of chin muscle tone shown in same color code as the brain waves. Links reflect the coupling strength between cortical rhythms at different locations and EMG frequency bands as quantified by the TDS measure (Methods 2.3). Color of the links correspond to the cortical rhythm involved in that particular interaction. Links strength is marked by line width — thin lines for 6% < *%TDS* < 12%, thick lines for *%TDS* > 12%. Shown are all links above a threshold *%TDS* = 6%. Sparser networks in PD subjects during light and deep sleep and thicker links during REM compared to healthy lead to a change in the sleep-stage stratification pattern. Overall, the differences between PD and healthy subjects are less pronounced in brain-chin interactions than brain-leg (Figure 2), showing a smaller loss of muscle atonia in chin compared to leg muscle tone during REM.

In the cortico-muscular network (Figure 2; Figure 4), brain areas are represented by Frontal (Fp1 and Fp2, F3 and F4), Central (C3 and C4) and Occipital (O1 and O2) EEG channels, where nodes with different color in each brain area represent distinct brain waves. Peripheral nodes indicate EMG frequency bands of leg (Figure 2) and chin (Figure 4) muscle tone shown in the same color code as the brain waves. Network links show the coupling strength of each cortical rhythm across cortical areas with an EMG frequency band (Rizzo et al., 2020). Links strength corresponds to the matrix elements in Figure 1A and Figure 3A, and is marked by line width: thin lines for 6% < *%TDS* < 9% for Wake, REM, and LS, 6% < *%TDS* < 8% for DS; thick lines for *%TDS* > 9% for Wake, REM, and LS, *%TDS* > 8% for DS for brain-leg network interactions (Figure 2); thin lines for 6% < *%TDS* < 12%, thick lines for *%TDS* > 12% for brain-chin network interactions for all sleep stages (Figure 4).

#### 2.5.2 Network of interactions between cortical rhythms and integrated EMG activity

**FIGURE 5:**
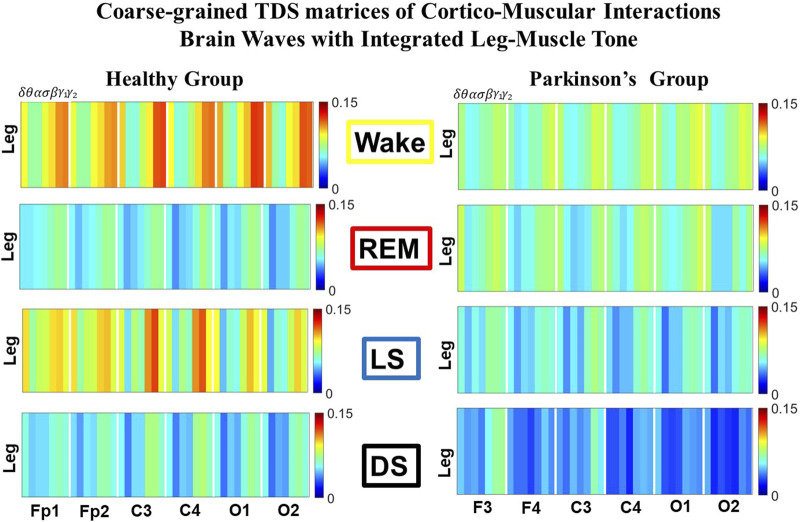
Dominant channels of communication and reorganization in brain-leg network interactions across physiological states in healthy and Parkinson’s subjects. Group-averaged matrices of coupling strength (measured as *%TDS*; see Methods 2.3) for brain vs. leg muscle tone interactions coarse-grained to represent the average coupling of each brain rhythm at a given cortical location with integrated spectral power of all leg EMG frequency bands for healthy (left panels), and PD subjects (right panels). Brain-leg networks exhibit pronounced reorganization with transition across sleep stages for both physiologic and pathologic conditions. Physiologic brain-leg networks show stronger coupling during Wake and LS, and weaker coupling during REM and DS, while PD networks exhibit a gradual decline in link strength from Wake to REM, LS and DS. Moreover, for each sleep stage, high frequency cortical rhythms exhibit stronger TDS coupling across all cortical areas (EEG channels) in healthy subjects (marked by warm colors); in PD subjects the role of main mediators in brain-leg networks interactions is equally played by high frequency cortical rhythms (*γ*_1_ and *γ*_2_ brain waves) as well as *δ* brain wave.

To obtain information on the relative contribution of each brain rhythm on a given EEG channel with the integrated EMG activity, we consider the average coupling strength of a given brain wave from a given EEG channel with all EMG bands (Rizzo et al., 2020). We coarse-grain the matrices in Figure 1A and Figure 3A by taking the average of the matrix elements along a given column, which means the average coupling of the integrated EMG activity with each cortical rhythm Δ*f*_*j*_, *j* = 1, … , 7 from a cortical location; the average is given by

$$n_{1h}=\frac{1}{7}\overset{7}{\underset{EMG\left(\Delta f_{i}\right):i=1}{\sum }}\bar{\%TDS}\left[EMG\left(\Delta f_{i}\right),Brain\left(\Delta f_{j}\right)\right]$$ (2)

where *h* = 7 (*k* − 1) + *j*, *k* = 1, … , 6 corresponding to a given EEG channel, and *%TDS* [*EMG* (Δ*f*_*i*_), *Brain* (Δ*f*_*j*_)] is the group-average %TDS between the frequency band Δ*f*_*i*_ of EMG and the cortical rhythm Δ*f*_*j*_ at a given EEG channel averaged across all subjects in each group (healthy in left panels and PD in right panels in Figure 5; Figure 8).

##### 2.5.2.1 Radar-chart graphical representation

**FIGURE 6:**
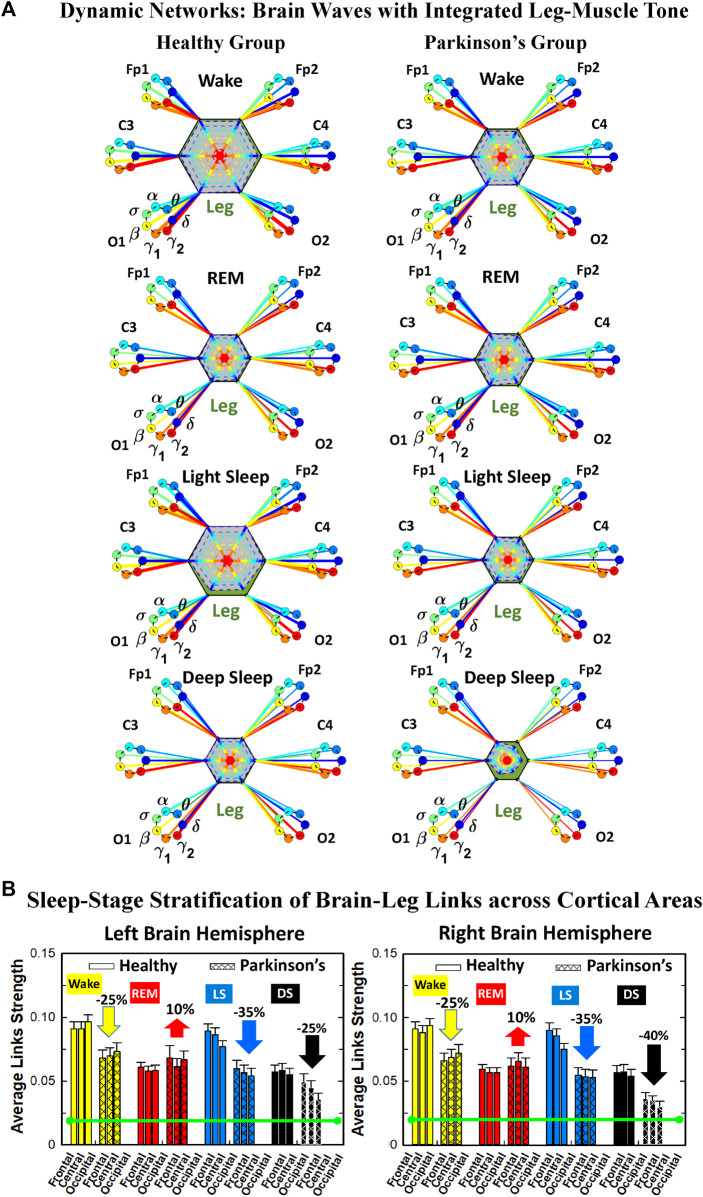
Dynamic networks of interaction between cortical rhythms and integrated leg-muscle tone across physiological states in healthy and Parkinson’s subjects. **(A)** Links in network maps represent group-averaged TDS coupling strength (Section Methods 2.5.1) between each brain rhythm at a given cortical location and the leg-muscle tone, after averaging over all leg EMG bands (see Section Methods 2.5.2), and correspond to the elements in the coarse-grained matrices shown in Figure 5 for healthy (left networks) and PD subjects (right networks). Brain areas are represented by Frontal, Central and Occipital EEG channels, and network nodes with different colors represent seven cortical rhythms (*δ*, *θ*, *α*, *σ*, *β*, *γ*_1_, *γ*_2_) for each EEG channel. Links strength is illustrated by line thickness, and links color corresponds to the color of brain rhythms (network nodes). Shown are all links with strength *%TDS* ≥ 2.3% corresponding to the significance threshold based on surrogate tests (Section Method 2.4). Radar-charts centered in the hexagons represent the relative contribution of brain control from different brain areas to the strength of network links during different sleep stages. The length of each segment along each radius in the radar-charts represents TDS coupling strength between each cortical rhythm at each EEG location and leg muscle tone. The segments are shown with the same color as the corresponding brain rhythms. The brain-leg network interactions are mainly mediated through high frequency *γ*_1_ and *γ*_2_ cortical rhythms (thicker orange and red links) in physiologic and pathologic conditions, and are characterized with relatively symmetric links strength to all six cortical areas, as shown by the symmetric radar-chart in each hexagon, with stronger contribution from the Frontal and Central areas. **(B)** Histograms of links strength in the brain-leg network during different sleep stages for healthy and PD subjects. Group-averaged links strength is obtained using the TDS measure, where each bar represents the average strength of interaction of all cortical rhythms from a given brain area in the left (left panel) and right brain hemisphere (right panel) with all muscle tone EMG bands. Error bars represent the standard error obtained for all subjects in each group; horizontal green lines in both panels mark a surrogate test threshold (*%TDS* = 2.3%; Section Method 2.4) above which network interactions are physiologically significant. Network reorganization is observed with transition across sleep stages: up-down-up-down for healthy, up-down-down-down for PD subjects.

We develop a radar-chart representation to map such interactions from across different brain areas (Figure 6A; Figure 9A). This network consists of i) six heptagons, one for each of the six brain areas corresponding to the locations of the EEG channels, and ii) a centered hexagon representing the leg (Figure 6A) or the chin (Figure 9A). Nodes in the heptagons are color-coded according to the following scheme: dark blue for *δ*, light blue for *θ*, turquoise for *α*, green for *σ*, yellow for *β*, orange for *γ*_1_ and red for *γ*_2_. Brain heptagons are connected to the organ hexagon by links whose thicknesses encode the corresponding coupling strengths (Rizzo et al., 2020). Networks include only links above a statistically significant threshold (Section 2.4). The radar-chart centered in the organ hexagon represents the relative contribution to muscle control from different brain areas. The length of each segment along each radius in the radar-charts represents TDS coupling strength between each cortical rhythm at each EEG channel location and leg (Figure 6A) or chin (Figure 9A) muscle tone.

#### 2.5.3 Network of interactions between EMG frequency bands and integrated EEG activity

Similarly, in order to obtain information on the relative contribution of each EMG frequency band on a given EMG muscle tone with the integrated EEG activity, we consider the average coupling strength of a given EMG frequency band with all brain waves from a given EEG channel (Rizzo et al., 2020). We coarse-grain the matrices in Figure 1A and Figure 3A by taking the average of the matrix elements along a given row, which means for each EMG frequency band Δ*f*_*i*_, *i* = 1, … , 7 the average coupling strength with the *k*th EEG channel is given by

$$m_{ik}=\frac{1}{7}\overset{7}{\underset{Brain\left(\Delta f_{j}\right):j=1}{\sum }}\bar{\%TDS}\left[EMG\left(\Delta f_{i}\right),Brain\left(\Delta f_{j}\right)\right].$$ (3)

##### 2.5.3.1 Graphical network representation of interactions between EMG frequency bands and integrated EEG activity

This type of network represents the response of a EMG band to signals from the brain. The focus is to understand the role of each EMG band in the brain-muscle communication, for instance if there is preferential EMG frequency, and if there is physiologic state specific pattern in the cross-talk (Figure 6; Figure 9). Each network is constituted by six heptagons representing the six EEG channels, whose spatial distribution reminds to the physical locations of electrodes on the brain surface from an axial point of view (Fp1/F3, C3 and O1 on the left side and Fp2/F4, C4, and O2 on the right side). Each of them represents the entire power spectrum of the corresponding EEG channel. The peripheral nodes represent the 7 frequency bands identified in the power spectrum of the leg (Figure 12) or chin (Figure 15) EMG muscle tone. The links between each node and a heptagon represent interactions of a given EMG band with each cortical location averaged over all cortical rhythms as defined in Eq. 3; color of links and nodes corresponds to the frequency bands (Rizzo et al., 2020). Only the links with a TDS ≥3% are plotted; the thickness depends on the coupling strength. In particular, there are three different types of link thickness: thin links with 4% (5%) ≤ TDS <6.5(9%)% , intermediate links with 6.5% (9%) ≤ TDS <8.5%(13%) , and thick links with TDS ≥8.5%(13%) for brain-leg (brain-chin) interactions.

### 2.6 Statistical tests

The following statistical test is used to validate the results on the comparison between healthy and Parkinson’s subjects: Wilcoxon test for pairwise comparisons. We note that the subjects that without a specific sleep stage in their recording (Data Section 2.1) are not considered in the corresponding statistical test. The statistical analysis is performed on MatLab.

## 3 Results

### 3.1 Dynamic network of cortical rhythms interactions with rhythms in muscle activity across sleep and wake in healthy and Parkinson’s subjects

We focus on the dynamics of cortico-muscular interactions, on their network structure and network reorganization during the sleep-wake cycle considering four states (Wake, REM, LS and DS) under healthy conditions, and investigate how these networks are affected by Parkinson’s. Brain activity is derived at six EEG channels, corresponding to six major cortical areas—frontal, central and occipital in the left (Fp1/F3; C3; O1) and right brain hemisphere (Fp2/F4; C4; O2) for healthy and Parkinson’s subjects. Muscle activity is measured from EMG channels, corresponding to leg and chin muscle tone. Simultaneous EEG and EMG signals are recorded during wake and sleep throughout the night period (Methods Section 2.1). Recorded signals at each brain and peripheral muscle location are decomposed into seven physiologically relevant frequency bands—*δ*, *θ*, *α*, *σ*, *β*, *γ*_1_, and *γ*_2_ — in order to understand the frequency-dependent brain-muscle cross-talk and to identify specific network communication pathways for each physiological state. We, therefore, consider a network of interactions, where at each cortical and muscle location we have seven network nodes representing physiological rhythms, which interact with all nodes across different cortical and muscle locations. Moreover, we investigate how the brain-muscle communication networks change structure and dynamics with major neurodegenerative disorders, such as PD.

We identify network interactions utilizing the TDS method (Methods Section 2.3), which is based on the concept of TDS (Bashan et al., 2012) and quantifies periods of stable time delay between synchronous bursts in cortical and muscle tone activity. Strong interactions (network links) correspond to long periods of stable time delay. Brain-leg and brain-chin interaction strength for each pair of cortical and muscular rhythms is shown in Figure 1 and Figure 3, and is represented by the TDS matrix elements for wake, REM, LS and DS (Figure 1A; Figure 3A). Bar charts in Figure 1B and Figure 3B show the average network links strength for healthy and PD subjects during each physiological state (Methods Section 2.5.1).

#### 3.1.1 Brain-leg interaction networks

We observe that brain-leg TDS interaction matrices in healthy subjects (left panels) reorganize with transition across sleep stages: coupling (links strength) is higher during Wake and LS, and lower during REM and DS (Figure 1), indicating stronger synchronization in bursting dynamics of cortical rhythms and leg muscle tone activity during Wake and LS, compared to REM and DS. Notably, the uncovered Wake-REM-LS-DS stratification pattern in network links strength for healthy subjects is significantly altered with PD, where a gradual decline in links strength is observed with transition from Wake to REM, to LS and DS. This change results from a dramatic ≈25% decrease in links strength during Wake, ≈25% decline during LS and ≈40% decline during DS, and an increase of ≈10% in links strength during REM for the PD group compared to healthy subjects. Wilcoxon test for pairwise comparisons of the average network links strength between the healthy and PD group for each brain hemisphere and each sleep stage (Figure 1B) shows a statistically significant difference during Wake, REM and LS (*p* ≤ 0.02) and borderline significant difference during DS (*p* = 0.11). Note that increased cortico-muscular coherence has been previously reported for subjects with idiopathic REM Behavior Disorder (Jung et al., 2012), but it has not been observed in PD subjects prior to this study. The uncovered sleep-stage differentiated response of cortico-muscular network interactions to Parkinson’s perturbation–i.e., significant decline in links strength during wake, LS and DS, vs. increase during REM–demonstrates the complexity of underlying mechanisms of motor control even at rest, and represents a new class of network-based biomarkers for early diagnosis of PD.

Further, the dynamic networks derived from the TDS method allow us to identify the cortical rhythms that play role of main mediators in the brain-muscle cross-talk for each sleep stage (strong coupling for pairs of cortical and muscle rhythms is indicated by warm colors in the TDS matrices, Figure 1A). In healthy subjects high frequency cortical rhythms, specifically *β*, *γ*_1_ and *γ*_2_, are involved in the strongest interactions with all leg EMG rhythms for all sleep stages (Figure 1A). In contrast, in PD subjects, the dominance of high frequency cortical rhythms is reduced, and an overall homogeneous contribution from all cortical rhythms to the brain-muscle network interactions is observed for wake and all sleep stages.

Remarkably, the observed sleep-stage stratification in group-average cortico-muscular network links strength is consistent for all individual subjects within the healthy and PD group, indicating an universal mechanism regulating cortico-muscular coordiantion (error bars in Figure 1B). Our findings demonstrate that there is a previously unrecognized complex network of synchronization among all cortical and muscular rhythms. This network is continuously present during a given sleep stage and follows a hierarchical reorganization with transition from one sleep stage to another (Figure 1). The observed differences in the sleep-stage stratification profile of brain-muscle networks between the healthy and PD group, and the changed role of main mediators in brain-muscle communication can be utilized as new early-stage biomakers of altered autonomic regulation of wake and sleep under PD.

Note that green lines in each bar plot (Figure 1B) indicate the physiological significance threshold, i.e. 2.3% TDS for coupling strength given by surrogate tests (Methods Section 2.4).

We next develop dynamic network representation of the information contained in the TDS matrices, where heptagons of nodes at different cortical locations represent cortical rhythms (EEG frequency bands), and peripheral nodes represent rhythms in muscle activity (EMG frequency bands) (Figure 2). Network links are derived from the TDS matrix elements and quantify the strength of pair-wise coupling among cortical and muscle rhythms (shown by links thickness). Links color corresponds to the cortical rhythms involved in the interaction.

We find that the obtained cortico-muscular networks undergo hierarchical reorganization with transition from wake to sleep and from one sleep stage to another. Specifically, for healthy subjects the brain-leg network is highly connected and has stronger links during Wake and LS, while network connectivity and links strength significantly declines during REM and DS (Figure 2). In contrast, the brain-leg network for the PD group undergoes a gradual decline in connectivity and links strength with transitions from Wake to DS. This reorganization of the brain-leg communication network results from the fact that different cortical and muscle rhythms play role of main mediators of brain-muscle interactions for the different states. We note that high-frequency *γ*_2_ cortical rhythms (red links) are involved in the strongest links for all sleep stages under healthy conditions, a behavior which is suppressed in the PD group. Comparing healthy vs. PD group for each sleep stage separately, we find that the brain-leg network for the PD group is characterized by lower connectivity and weaker links for all states, except for REM (Figure 2).

#### 3.1.2 Brain-chin interaction networks

Results for brain-chin interactions (Figure 3) are similar to brain-leg: the sleep-stage stratification pattern changes with PD. Brain-chin coupling in healthy subjects exhibits overall stronger links during Wake and LS, and weaker during REM and DS, while for PD subjects a gradual decline is observed in links strength from Wake to DS. This common feature between the two different muscle groups (leg and chin) marks a characteristic behavior that affects and reorganizes the brain-muscle communication networks across sleep stages. However, the change between healthy and PD in brain-chin links strength is smaller compared to brain-leg interactions: PD links exhibit a dramatic decrease of coupling strength during LS (20% for both hemispheres) and DS (10% for the left and 20% for the right hemisphere) compared to controls, and a moderate increase in links strength during REM (10% for the left and 5% for the right hemisphere). A Wilcoxon test for pairwise comparisons between links strength average in healthy and PD for each brain hemisphere and each sleep stage (Figure 3B) shows a statistically significant difference during REM and LS (*p* ≤ 0.04), and borderline significant difference during DS (*p* ≤ 0.26). We note that, comparing with brain-leg network interactions, in brain-chin interactions, there is not a significant decline in link strength during wake, and the decline during LS and DS is more moderate. This demonstrates that in brain-chin network interactions, the PD response of change to sleep-stage regulation is slightly different than brain-leg interactions: we have a more moderate change compared to brain-leg interactions, where the decrease of link strength arrives to 40% (Figure 1B). Thus, the cortico-muscular regulation may be muscle specific in sleep.

Furthermore, we observe that specific cortical rhythms have a dominant role in the brain-chin communication depending on the state. Specifically, in healthy subjects during Wake *γ*_1_ and *γ*_2_ mediate the strongest brain-chin interactions with all chin EMG frequency bands (Figure 3A). High frequency cortical rhythms involve strong links also during REM, LS and DS, where they share dominant role in cortico-muscular communication with the low frequency cortical rhythms (*δ*, *θ*, *α*) which exhibit stronger interaction with the same frequency bands of chin EMG (warmer colors for the diagonal elements in the TDS matrix blocks, Figure 3A). In contrast, in PD subjects only high frequency cortical rhythms play dominant role in the brain-chin cross-talk during all sleep stages. Notably, during DS both healthy and PD groups are characterized by stronger interactions between identical frequency bands in the cortex and in chin muscle tone (warmer colors for the diagonal TDS elements). The observed brain-chin interaction patterns in Figure 3A are absent in the brain-leg interactions (Figure 1A) for both healthy and PD subjects during REM, LS and DS—a difference which may relate to the different architecture of chin and leg muscles, different histochemical composition of their muscle fibers, and different ratio fast/slow motor units in these muscles. Our findings demonstrate a previously unrecognized dependence of cortico-muscular communication on physiologic states such as sleep, sleep stages, and resting wake in the absence of targeted movements, and indicate that cortico-muscular control is significantly modulated by the mechanisms of autonomic regulation.

As for the brain-leg networks in Figure 2, we map the brain-chin TDS matrices into networks where nodes represent all brain EEG and chin muscle EMG frequency bands and links show the degree of their pair-wise coupling (Figure 4). Our analysis show that for healthy subjects both brain-leg and brain-chin networks undergo complex reorganization across sleep stages with stronger links during wake and LS, and weaker links during REM and DS. In contrast, for PD subjects there is a gradual decline in network connectivity and links strength with transition from wake to REM, to LS and DS, that, in comparison to healthy subjects, is characterized by higher connectivity and links strength during REM and network breakdown during DS. We note that the effect of PD on cortico-muscular interactions is less pronounced for the brain-chin network (i.e., less decline in connectivity and links strength) compared to the brain-leg network. Further, for each sleep stage the hierarchical organization of cortico-muscular networks is characterized by clusters of strong links representing the coupling for specific pairs of cortical rhythms at given brain areas and chin EMG frequency bands that play role as main mediators of the interactions. In both healthy and PD group the strongest network links correspond to interactions involving high-frequency *γ*_1_ and *γ*_2_ cortical rhythms (marked by orange and red thick links) for all sleep stages. In addition, low frequency cortical rhythms *δ*, *θ*, *α* (dark and light blue thick links) involve strong interactions during REM for healthy and during DS for PD subjects (Figure 4).

The established reorganization of the brain-muscle interaction networks with transition across sleep stages demonstrates that a particular set of strong links, representing the interaction between specific cortical and muscle rhythms, plays role as main mediators in brain-muscle control. We find that each physiological state (wake, sleep stages) is associated with a unique network structure with a specific profile of distribution network links strength, and that the network organization for all states changes with neurodegenerative disorder, such as Parkinson’s.

### 3.2 Dynamic networks of cortical rhythms interactions with integrated muscle activity across sleep and wake in healthy and Parkinson’s subjects

#### 3.2.1 Network interactions of distinct cortical rhythms and integrated leg-muscle tone

To understand the role cortical rhythms at different cortical locations play in muscle control during resting wake and sleep stages, we consider the coarse-grained TDS matrix for each state, where columns in each block of the matrix represent the average coupling of a given cortical rhythm (horizontal axis) with all rhythms embedded in the leg muscle-tone (Figure 5). In the following text these averaged coarse-grained matrices for each state are referred as brain-to-muscle interaction matrices for the healthy and PD group. We find that each physiological state is associated with a specific structure of the coarse-grained interaction matrix, and that this matrix undergoes complex reorganization with transition across states—behavior observed for both healthy and PD subjects, although with a different pattern of sleep-stage stratification in coupling strength. Specifically, the brain-leg matrix for healthy subjects shows stronger coupling during Wake and LS, and weaker coupling during REM and DS, with pronounced interactions of high-frequency cortical rhythms with integrated muscle activity consistently for all states. This sleep-stage stratification pattern is disrupted in PD subjects, where the strength of interaction is significantly reduced for all cortical rhythms, and shows a gradual decline from wake to REM, LS, and DS.

To visualize information present in the coarse-grained TDS matrices (Figure 5) we utilize radar-charts graphical representation to derive the brain-to-leg networks in Figure 6A PD interaction networks clearly show an overall decrease in link strength compared to healthy (general smaller size of the leg hexagon in PD networks). Observing in more details the dynamic change of the radar-chart size and structure across sleep stages, we note different patterns for healthy and PD subjects. The uncovered structure of the brain-to-muscle interaction matrix for healthy subjects during wake and sleep (Figure 5) demonstrates that high frequency cortical rhythms have dominant role in cortico-muscular control for all sleep stages. In contrast, in PD subjects both high-frequency cortical rhythms (*γ*_1_ and *γ*_2_) as well as the low-frequency *δ* rhythm are main mediators of brain-leg interactions. Brain-to-leg interaction networks (Figure 6A) exhibit a general symmetry between left and right brain hemisphere and among different cortical locations across all sleep stages for both healthy and PD subjects. Further, in healthy subjects we find stronger brain-leg interactions for the frontal and central cortical areas during LS—behavior not observed in PD subjects (Figure 6B). In contrast, more pronounced brain-leg interactions in PD subjects are observed for the frontal and central left brain areas during DS (Figure 6B).

**FIGURE 7:**
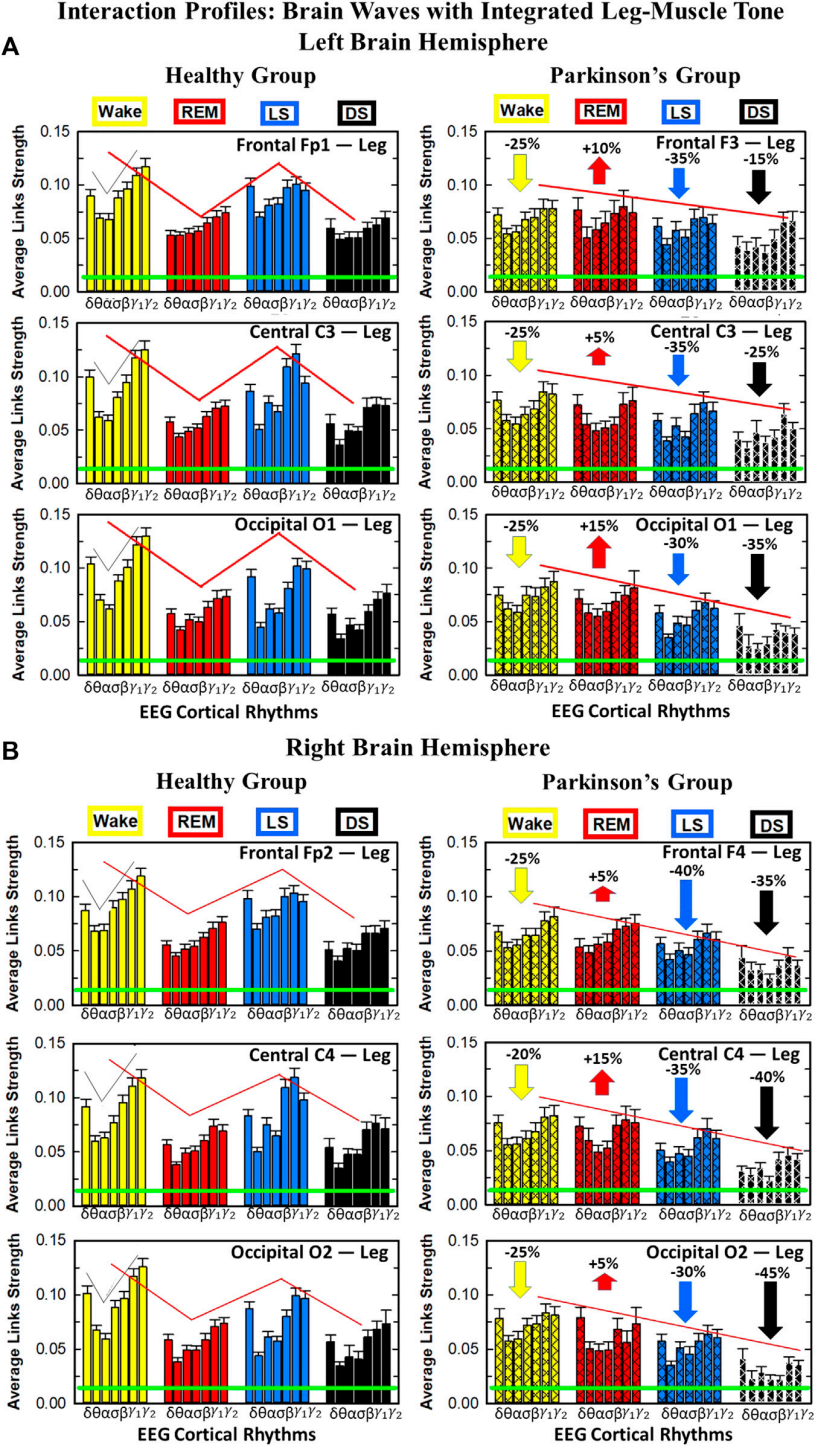
Characteristic profiles of network links strength for cortical rhythms interactions with integrated leg-muscle tone in healthy and Parkinson’s subjects. Group averaged links strength are obtained using the TDS measure, where each link represents the interaction of the leg-muscle tone (averaged over all EMG bands, as in Figure 5) with each cortical rhythm at a given brain area in the left **(A)** or right **(B)** brain hemisphere for healthy (left panels) and PD subjects (right panels). Links are grouped by sleep stage and brain areas, and are ordered from low-to high-frequency cortical rhythms for each sleep stage. Groups of bar charts represent network links between nodes (cortical rhythms) in each brain location and the radar-charts (sum of interactions with all leg EMG bands) as shown in Figure 6A. Note that links presented as groups of bar charts in each panel correspond to 1 bar in the histograms of averaged links strength for each brain area shown in Figure 6B. Error bars represent the standard error obtained for all subjects in each group; horizontal green lines in both panels mark a surrogate test threshold (*%TDS* = 2.3%; Section Method 2.4) above which network interactions are physiologically significant. The Wake-REM-LS-DS alternation of healthy subjects is absent in PD, where an enhanced REM link strength - 5% − 15% for both hemispheres (*p* ≤ 10^−3^ Wilcoxon test for left **(A)**, and *p* ≤ 0.0034 Wilcoxon test for right hemisphere **(B)**) - and a decrease of coupling strength during Wake, LS and DS - 15% − 35% for left hemisphere and 20% − 40% for right hemisphere (statistically significant difference – *p* ≤ 0.0272 Wilcoxon test for left and *p* ≤ 0.0299 Wilcoxon test for right hemisphere–during Wake and LS and borderline significant during DS) are observed compared to healthy. This behavior may be pathognomonic for patients with PD suggestive of increased muscle tone during REM and decreased muscle tone during NREM sleep stages, as a result of cortical-leg dissociation and decreased normal inhibition of tone in REM phase. A characteristic profile of network links strength as function of cortical rhythms frequency is observed for healthy subjects, while PD network links exhibit a different frequency profile. This change in PD is mostly due to a decrease of network link strength for *β*, *γ*_1_ and *γ*_2_ brain waves during Wake and NREM sleep stages, and an increase of link strength for *δ* and *θ* brain waves during REM. In both groups of subjects, the profile is robust, with almost identical shape across different brain areas for both hemispheres.

The effect of Parkinson’s on cortico-muscular networks across sleep stages can follows two possible scenarios: i) PD affects the links strength homogenously across brain waves, or ii) PD affects the brain-muscle communication in a complex way, preferring certain cortical rhythms more than others, i.e., affecting the link strength of interactions involving particular brain waves more than others. In order to understand how PD changes the cortico-muscular networks structure, we study the characteristic network links strength profile of interactions between individual cortical rhythms at a given brain location and integrated leg EMG activity (Figure 7) for healthy (left panels) and PD subjects (right panels) across different sleep stages. The change in sleep-stage stratification pattern with PD, already observed in the previous figures, is now investigated in more details: i) the percentage of increase/decrease in link strength is correspondingly given for each brain location, ii) the particular change in link strength for each interaction involving individual cortical rhythms and integrated leg muscle tone activity is now presented. During Wake almost all brain locations exhibit the same decline in link strength (25% for all brain areas, except for C4 with 20% decline), while during LS frontal F3 and F4 EEG locations show the highest decrease (35% and 40% respectively–Figures 7A,B), central areas a medium decrease (35% for both hemispheres), and occipital areas the smallest decrease (30% for both hemispheres). Differently, during DS the percentage of decrease across different brain locations follows the opposite pattern: smallest decrease for frontal areas (15% for left and 35% for right hemisphere), medium for central areas (25% for left and 40% for right hemisphere), and highest for occipital areas (35% for left and 45% for right hemisphere). The right hemisphere is overall more affected by Parkinson’s in the breakdown of link strength of brain-leg muscle interactions. The moderate increase in link strength in PD compared to healthy during REM follows opposite patterns across brain locations in left and right hemisphere: central left C3 EEG location registers the smallest increase in link strength 5% compared to F3 and O1, while the right central C4 exhibit the highest increase 15% compared to F4 and O2. A Wilcoxon test for pairwise comparisons between links strength average in healthy and PD for each left brain location and each sleep stage (Figure 7A) shows a statistically significant difference during Wake, REM and LS (*p* ≤ 0.03), and borderline significant difference during DS (0.03 ≤ *p* ≤ 0.28). Similarly, for the right hemisphere (Figure 7B) the pairwise comparisons test between healthy and PD shows a statistically significant difference during Wake, REM and LS (*p* ≤ 0.03), and borderline significant difference during DS (0.12 ≤ *p* ≤ 0.72).

We find that for a given sleep stage the interaction profile of links strength in the brain-to-leg network is consistent across all cortical areas in the left and right hemispheres for both healthy and PD subjects. Further, different sleep stages are characterized by different links strength interaction profiles (marked by different colors in Figure 7), indicating a unique brain-leg network structure and dynamics for each physiological state. However, the profile dramatically changes with Parkinson’s, providing new information on the effect of PD on individual cortical rhythms interactions: not only PD causes a general change in link strength (increase during REM and decrease during Wake and NREM sleep stages), but also it affects the different cortical rhythms in a different way, altering the frequency profile observed in physiologic conditions. Healthy brain-to-leg interactions profile is characterized by strongest links for the high-frequency bands *γ*_1_ and *γ*_2_ and a gradual decrease in links strength for the lower-frequency bands *β*, *σ*, *α*, *θ* followed by a slight kink up in link strength for the *δ* band. This characteristic profile is very marked during Wake, and gradually flattens during REM, LS and DS, keeping though the general shape. The frequency profile in PD is similar but not identical. The role of main mediators in brain-leg interactions played for healthy subjects by high-frequency bands, is here shared, during Wake, and almost overtaken, during REM, LS and DS, by the lowest frequency band *δ*. Moreover, the ratio between the link strength of interactions involving high-frequency cortical rhythms and medium-frequency cortical rhythms is lower for PD than for healthy. The change in the frequency profile during Wake, LS and DS, sleep stages that register a decrease in link strength, is due to a main effect of PD on high-frequency bands *γ*_1_ and *γ*_2_, main mediators in brain-leg interactions in physiologic conditions, while during REM, where an increase in link strength is observed, PD mainly affects *δ* band.

Within the current theoretical framework basic physiological states are traditionally identified and characterized by the dynamics of individual systems (e.g., brain or muscle tone) and by the presence of dominant EEG cortical rhythms or change in the EMG amplitude of muscle tone (e.g., DS with dominant EEG-*δ*, LS with dominant EEG-*θ*, REM with dominant EEG-*α* and suppressed EMG amplitude, *etc.*). Our empirical analyses demonstrate identifying and quantifying the network of interactions between physiological systems and the distinct rhythms embedded in their dynamics is essential to fully describe physiological states, understand the underlying regulatory mechanisms and assess perturbation due to pathological disorders, such as Parkinson’s. We find that in healthy conditions the network structure of cortico-muscular communications reorganizes across sleep stages, and uniquely defines a given physiologic state. Moreover, for the first time we uncover physiologic laws of regulation in muscle control in rest, during sleep, in absence of targeted movements, and their collapse with Parkinson’s. The dramatic change of the network reorganization pattern in the sleep-stage regulation is investigated in very details, showing not only a general effect of PD on network links strength, but also a complex mechanism that sees particular frequency bands as the main target of PD in cortico-muscular communications and their reorganization across sleep stages.

#### 3.2.2 Network interactions of distinct cortical rhythms and integrated chin-muscle tone

**FIGURE 8:**
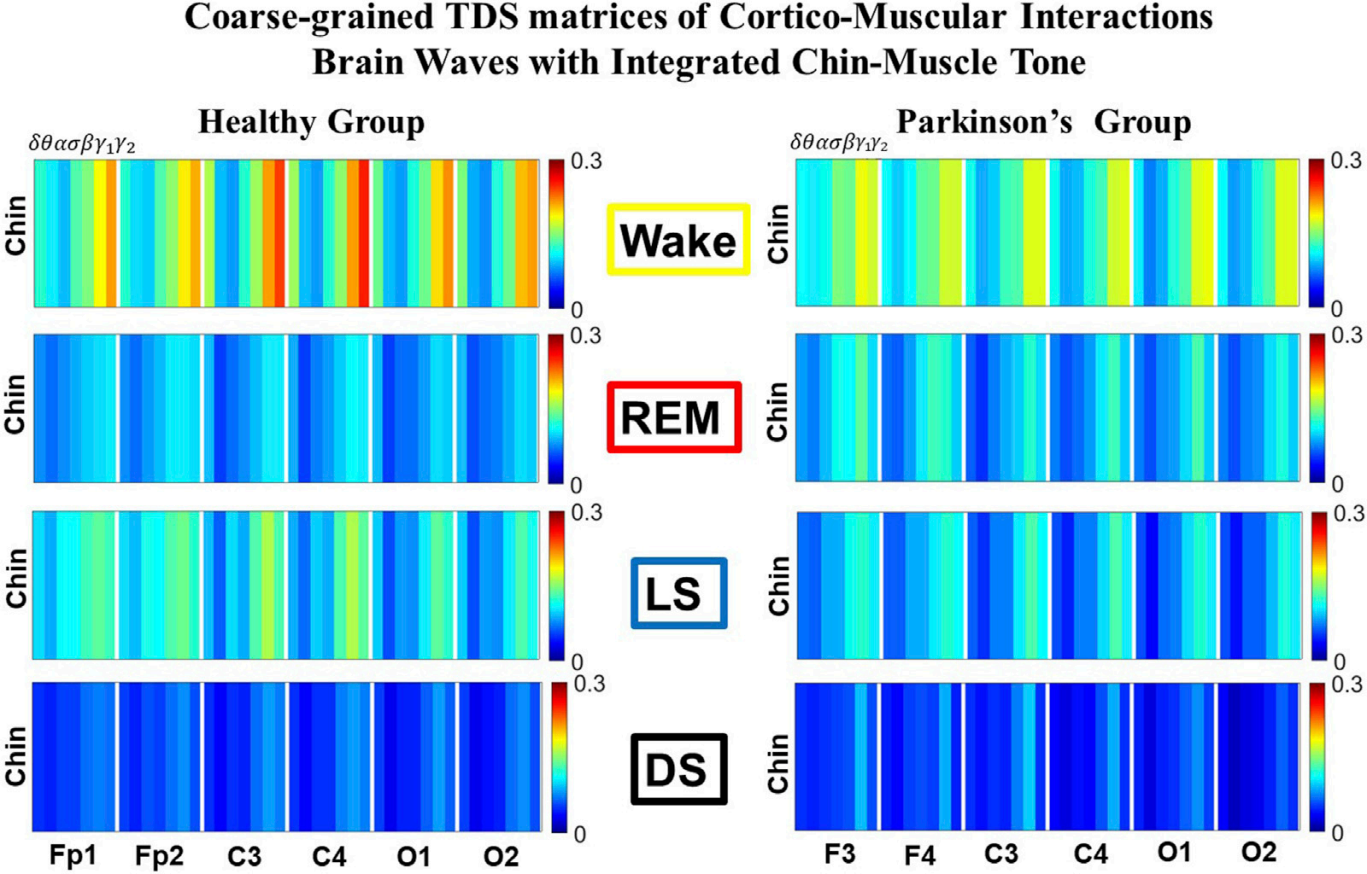
Dominant channels of communication and reorganization in brain-chin network interactions across physiological states in healthy and Parkinson’s subjects. Group-averaged matrices of coupling strength (measured as *%TDS*; see Methods 2.3) for brain vs. chin muscle tone interactions coarse-grained to represent the average coupling of each brain rhythm at a given cortical location with integrated spectral power of all chin EMG frequency bands for healthy (left panels), and PD subjects (right panels). Brain-chin networks exhibit pronounced reorganization with transition across sleep stages for both physiologic and pathologic conditions. Physiologic brain-chin networks show stronger coupling during Wake and LS, and weaker coupling during REM and DS, while PD networks exhibit a gradual decline in link strength from Wake to REM, LS and DS. Moreover, for each sleep stage, high frequency cortical rhythms exhibit stronger TDS coupling across all cortical areas (EEG channels) in healthy as well as in PD subjects (marked by warm colors).

**FIGURE 9:**
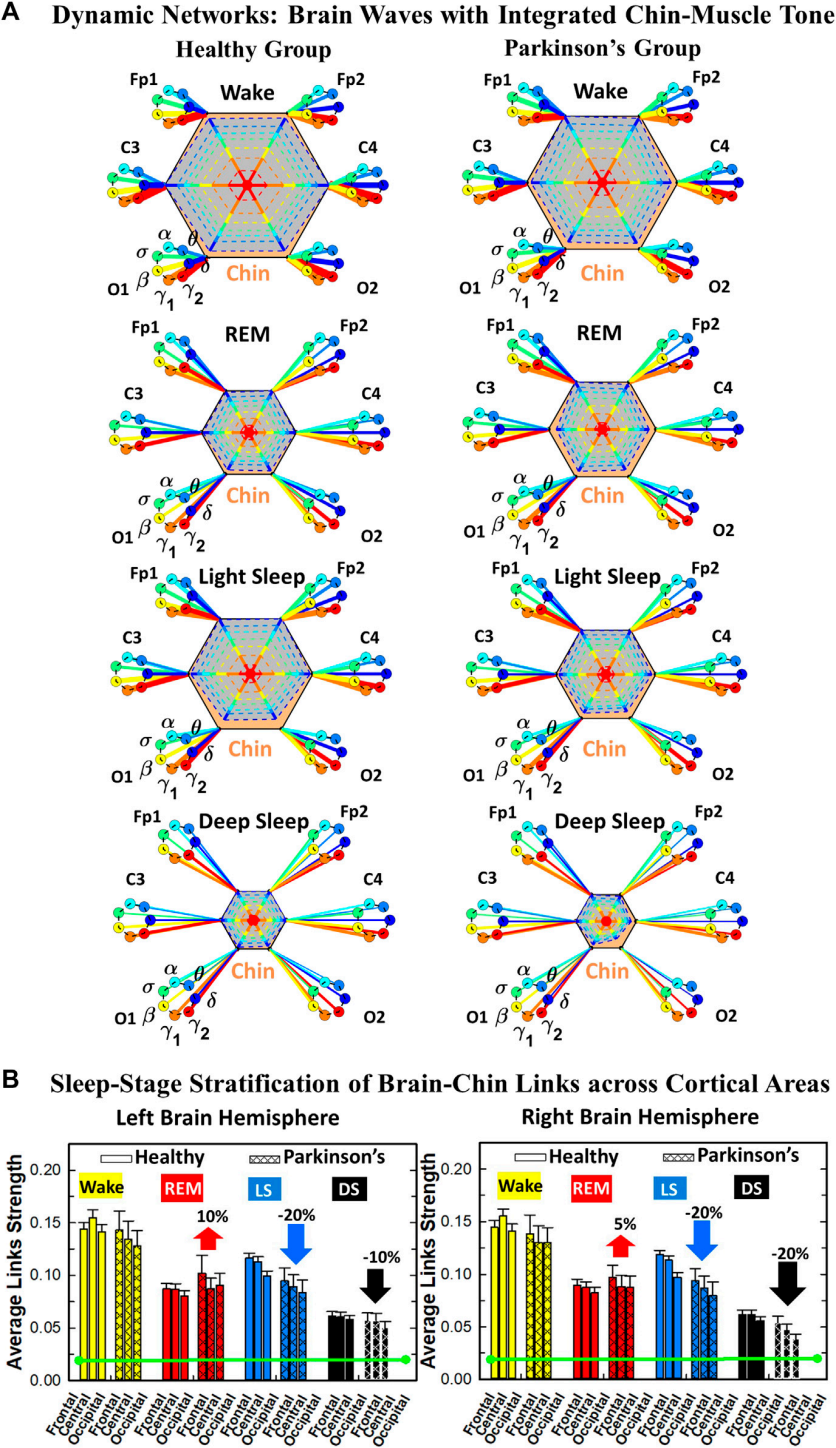
Dynamic networks of interaction between cortical rhythms and integrated chin-muscle tone across physiological states in healthy and Parkinson’s subjects. **(A)** Links in network maps represent group-averaged TDS coupling strength (Section Methods 2.5.1) between each brain rhythm at a given cortical location and the chin-muscle tone, after averaging over all chin EMG bands (see Section Methods 2.5.2), and correspond to the elements in the coarse-grained matrices shown in Figure 8 for healthy (left networks) and PD subjects (right networks). Brain areas are represented by Frontal, Central and Occipital EEG channels, and network nodes with different colors represent seven cortical rhythms (*δ*, *θ*, *α*, *σ*, *β*, *γ*_1_, *γ*_2_). Links strength is illustrated by line thickness, and links color corresponds to the color of brain rhythms (network nodes). Shown are all links with strength *%TDS* ≥ 2.3% corresponding to the significance threshold based on surrogate tests (Section Method 2.4). Radar-charts centered in the hexagons represent the relative contribution of brain control from different brain areas to the strength of network links during different sleep stages. The length of each segment along each radius in the radar-charts represents TDS coupling strength between each cortical rhythm at each EEG location and chin muscle tone. The segments are shown with the same color as the corresponding brain rhythms. The brain-chin network interactions are mainly mediated through high frequency *γ*_1_ and *γ*_2_ cortical rhythms (thicker orange and red links) in physiologic and pathologic conditions, and are characterized with relatively symmetric links strength to all six cortical areas, with stronger contribution from the Frontal (healthy during REM and LS) and Central areas. **(B)** Histograms of links strength in the brain-chin network during different sleep stages for healthy and PD subjects. Group-averaged links strength is obtained using the TDS measure, where each bar represents the average strength of interaction of all cortical rhythms from a given brain area in the left (left panel) and right brain hemisphere (right panel) with all muscle tone EMG bands. Error bars represent the standard error obtained for all subjects in each group; horizontal green lines in both panels mark a surrogate test threshold (*%TDS* = 2.3%; Section Method 2.4) for physiological significance. Network reorganization is observed with transition across sleep stages: up-down-up-down for healthy, up-down-down-down for PD subjects.

To investigate how rhythms at different cortical locations are involved in muscle control during wake and sleep, we next coarse-grain the TDS matrix for each physiological state where columns in each block of the matrix represent the average coupling of a given cortical rhythm (horizontal axis) with all rhythms embedded in the chin muscle-tone (Figure 8). We utilize radar-charts graphical representation to map the interaction of cortical rhythms with integrated chin muscle tone (Figure 9A) from the coarse-grained brain-chin TDS matrices in Figure 8. The average links strength for the left and right brain hemisphere sub-networks and for modules within each sub-network are shown in Figure 9B. With transition across sleeps stages the coarse-grained brain-chin interaction matrix undergoes structural reorganization with a stratification pattern, similar to the one observed for the coarse-grained brain-leg matrix. We find that coarse-grained brain-chin network for healthy subjects exhibits an up-down-up-down sleep-stage stratification pattern, with stronger links during Wake (thicker lines), weaker during LS, and weakest coupling during REM and DS (thinner lines). In contrast, the brain-chin network for PD subjects exhibits a very different transition with sleep stages characterized by an up-down-down-down pattern, with a gradual decline in link strength from Wake to DS (Figure 9A). This sleep-stage stratification in the brain-chin network structure is presented in more detail in Figure 9B, where bars represent the average links strength for different network modules (frontal, central and occipital) in the left and right brain hemispheres sub-networks.

The brain-to-muscles interaction matrices for both healthy and PD subjects (Figure 8) clearly show the dominance of high frequency cortical rhythms across all sleep stages, demonstrating that this general physiological law in chin muscle control is robust and invariant to pathologic conditions. We find that PD effect on brain-chin interaction networks is different from brain-leg networks, demonstrating that the muscle control in both physiologic and pathologic conditions depends on the particular muscle group analyzed. We find a left- and right-brain hemisphere symmetry in the interaction network of cortical rhythms with integrated chin muscle tone, which is consistent for all sleep stages (Figure 9. Moreover, the average network links strength of network modules corresponding to different cortical areas within each brain hemisphere exhibits a non-uniform pattern for both healthy and PD subjects, with a prevalence in links strength for cortical rhythms from central areas (C3 and C4) during Wake for healthy, and frontal areas (Fp1 and Fp2) for healthy during REM and LS and (F3 and F4) for PD across all sleep stages.

**FIGURE 10:**
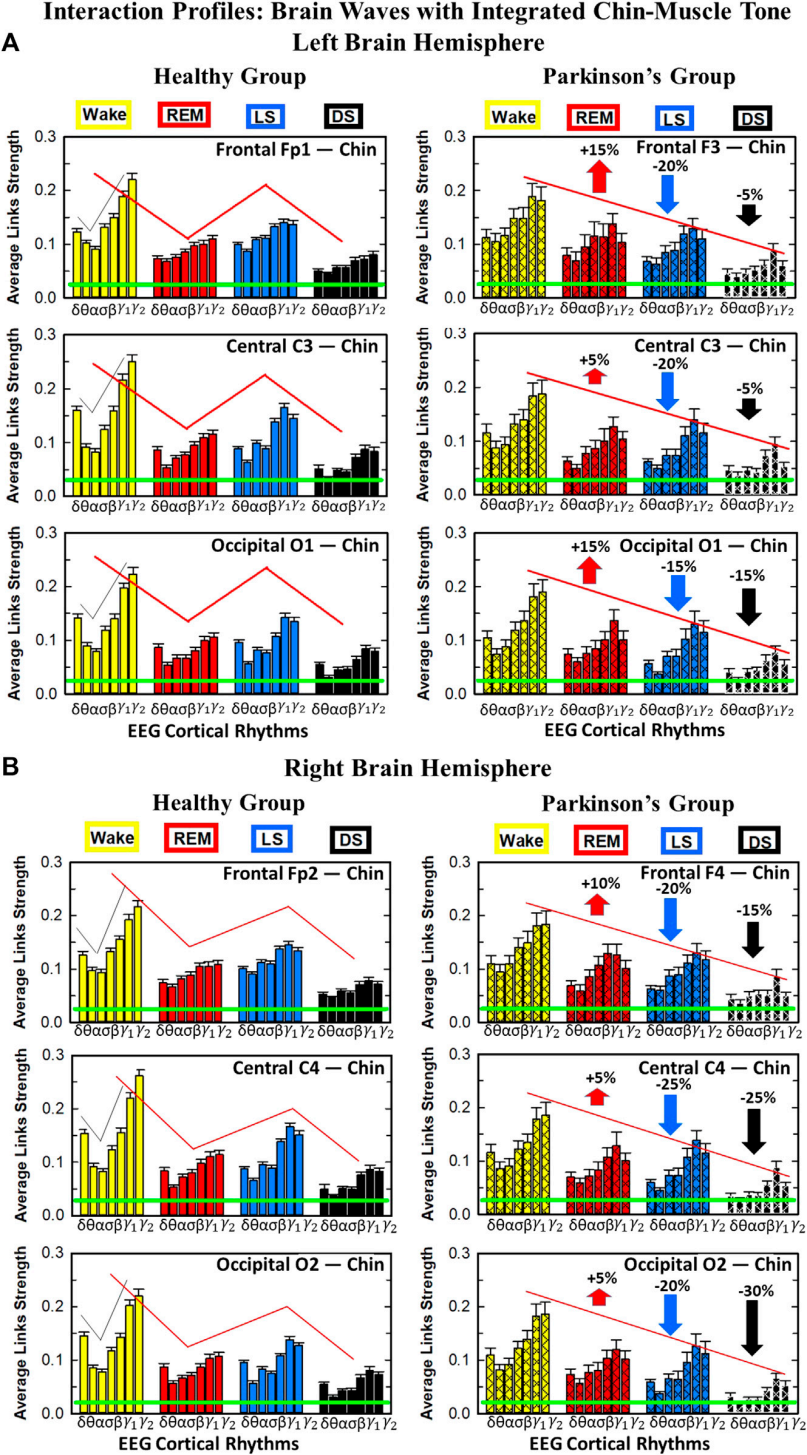
Characteristic profiles of network links strength for cortical rhythms interactions with integrated chin-muscle tone in healthy and Parkinson’s subjects. Group averaged links strength are obtained using the TDS measure, where each link represents the interaction of the chin-muscle tone (averaged over all EMG bands, as in Figure 8) with each cortical rhythm at a given brain area in the left **(A)** or right **(B)** brain hemisphere for healthy (left panels) and PD subjects (right panels). Links are grouped by sleep stage and brain areas, and are ordered from low-to high-frequency cortical rhythms for each sleep stage. Groups of bar charts represent network links between nodes (cortical rhythms) in each brain location and the radar-charts (sum of interactions with all chin EMG bands) as shown in Figure 9A. Note that links presented as groups of bar charts in each panel correspond to 1 bar in the histograms of averaged links strength for each brain area shown in Figure 9B. Error bars represent the standard error obtained for all subjects in each group; horizontal green lines in both panels mark a surrogate test threshold (*%TDS* = 2.3%; Section Method 2.4) above which network interactions are physiologically significant. The reorganization of cortico-muscular networks with transitions across sleep stages is perturbed with PD, leading to a different sleep-stage stratification pattern. Brain-chin interactions in PD show a decrease of 5% − 20% in the left hemisphere **(A)** and 15% − 30% in the right hemisphere **(B)** during light and deep sleep (statistically significant difference – *p* ≤ 0.0260 Wilcoxon test in the left and *p* ≤ 0.0164 Wilcoxon test in the right hemisphere – during LS, with exception of O1 – *p* = 0.0939 Wilcoxon test – and borderline significant during DS) and an increase of 5% − 15% and 5% − 10% during REM for left and right hemisphere respectively (*p* ≤ 0.0208 Wilcoxon test for the left and *p* ≤ 0.0089 Wilcoxon test for the right hemisphere). A characteristic profile of network links strength as function of cortical rhythms frequency is observed for healthy subjects. PD network links exhibit a similar frequency profile with a particular decrease of link strength for low frequency cortical rhythms *δ* and *θ* during LS. In both groups of subjects, the profile is robust, exhibiting almost identical shape across different brain areas in both hemispheres.

Further, we are interested to understand the complex mechanism of PD effect on muscle control across the whole spectrum of different cortical rhythms at different cortical areas. Therefore, we analyze the profile of network links strength of interactions between individual cortical rhythms at a given brain location and integrated chin EMG activity (Figure 10) for healthy (left panels) and PD subjects (right panels). Similarly to brain-leg interaction networks, Figure 10 provides new information not only on the sleep-stage reorganization of the cortico-muscular network in healthy conditions, but also on the particular change of links strength with PD at each brain area and for each cortical rhythm. Comparing the percentage decrease of link strength with PD during LS and DS across cortical areas we note that the two sleep stages exhibit different pattern, but generally symmetric between left and right hemispheres. Frontal F3 and central C3 left EEG locations show the highest decrease 20% during LS, while the occipital O1 left EEG location the smallest decrease 15% (Figure 10A); similarly, on the right hemisphere central C4 right EEG location show the highest decrease 25%, and frontal F4 and occipital O2 the smallest decrease 20% (Figure 10B). On the other hand, during DS both left and right hemispheres show the opposite pattern across cortical areas: smallest decrease for frontal areas (5% for left and 15% for right hemisphere), medium for central areas (5% for left and 25% for right hemisphere), and highest for occipital areas (15% for left and 30% for right hemisphere). The right hemisphere is overall more affected by Parkinson’s in a breakdown of link strength of brain-chin muscle interactions, as for brain-leg interactions. Regarding the increase in link strength during REM the frontal areas F3 and F4 register the highest increase (15% for left and 10% for right hemisphere), compared to a milder increase in central areas (5% for both hemispheres) and occipital (15% for left and 5% for right hemisphere), following the pattern observed during LS. Differently to the decrease in brain-chin link strength with PD during LS and DS that affected more the right hemisphere, the increase in link strength during REM is overall more present in the left hemisphere. A Wilcoxon test for pairwise comparisons between links strength average in healthy and PD for each brain location and each sleep stage shows a statistically significant difference (*p* ≤ 0.02 for all brain areas during REM and LS, with exception of O1 during LS *p* = 0.09), and borderline significant difference during DS (*p* = 0.09, *p* = 0.12 and *p* = 0.47 for left hemisphere, and *p* = 0.11, *p* = 0.17 and *p* = 0.61 for right hemisphere).

We note that for each sleep stage the links strength profile preserves the same shape for all network modules representing interactions of cortical rhythms at the frontal, central and occipital areas with integrated chin muscle tone. This is consistently observed for both healthy and PD subjects, although the shape of the links strength profiles is modulated with PD. Differently from brain-to-leg interactions network, the profile remains stable also with Parkinson’s, exhibiting almost identical shape compared to healthy networks during all sleep stages and across all brain areas. Similarly to brain-leg interactions healthy brain-to-chin interactions profile is characterized by strongest links for the high-frequency bands *γ*_1_ and *γ*_2_ and a gradual decrease in links strength for the lower-frequency bands *β*, *σ*, *α*, *θ* followed by a slight kink up in link strength for the *δ* band. This characteristic profile is very marked during Wake, and gradually flattens during REM, LS and DS, keeping though the general shape. The frequency profile in PD is almost identical, as mentioned before, with the only difference that *δ*-frequency brain wave interactions have generally the same link strength as for the medium-frequency brain waves interactions. We note that Parkinson’s affects brain-chin interactions in a different way compared to brain-leg interactions.

Our findings demonstrate that the cortico-muscular interaction networks dramatically reorganize in PD subjects with significant decline in network link strength during LS and DS and increase in interaction strength during REM, leading to breakdown in the “up-down-up-down” sleep-stage stratification pattern observed for the healthy group (Figure 6; Figure 9). While network links are significantly stronger in the brain-chin network compared to the brain-leg network in healthy subjects, both networks exhibit similar sleep-stage stratification, indicating universal behavior in muscle autonomic control depending on the physiological state under healthy condition. In contrast, for Parkinson’s we find a differentiated effect on cortico-muscular interactions depending on the muscle type with i) more significant decline of links strength in the brain-leg network across cortical areas for wake, LS and DS, and more significant increase in links strength during REM, compared to the brain-chin network; and ii) muscle-specific modulation in the links strength interaction profiles with significant decline in *γ*_1_-and *γ*_2_-mediated links for the brain-leg network across cortical locations and physiological states, compared to the brain-chin network where the decline is homogeneous for all network links preserving the shape of the interaction profiles (Figure 7; Figure 10).

### 3.3 Dynamic networks of integrated activity at different cortical locations with rhythms in muscle activity across sleep and wake in healthy and Parkinson’s subjects

#### 3.3.1 Network interactions of integrated cortical activity with distinct rhythms in leg-muscle tone

**FIGURE 11:**
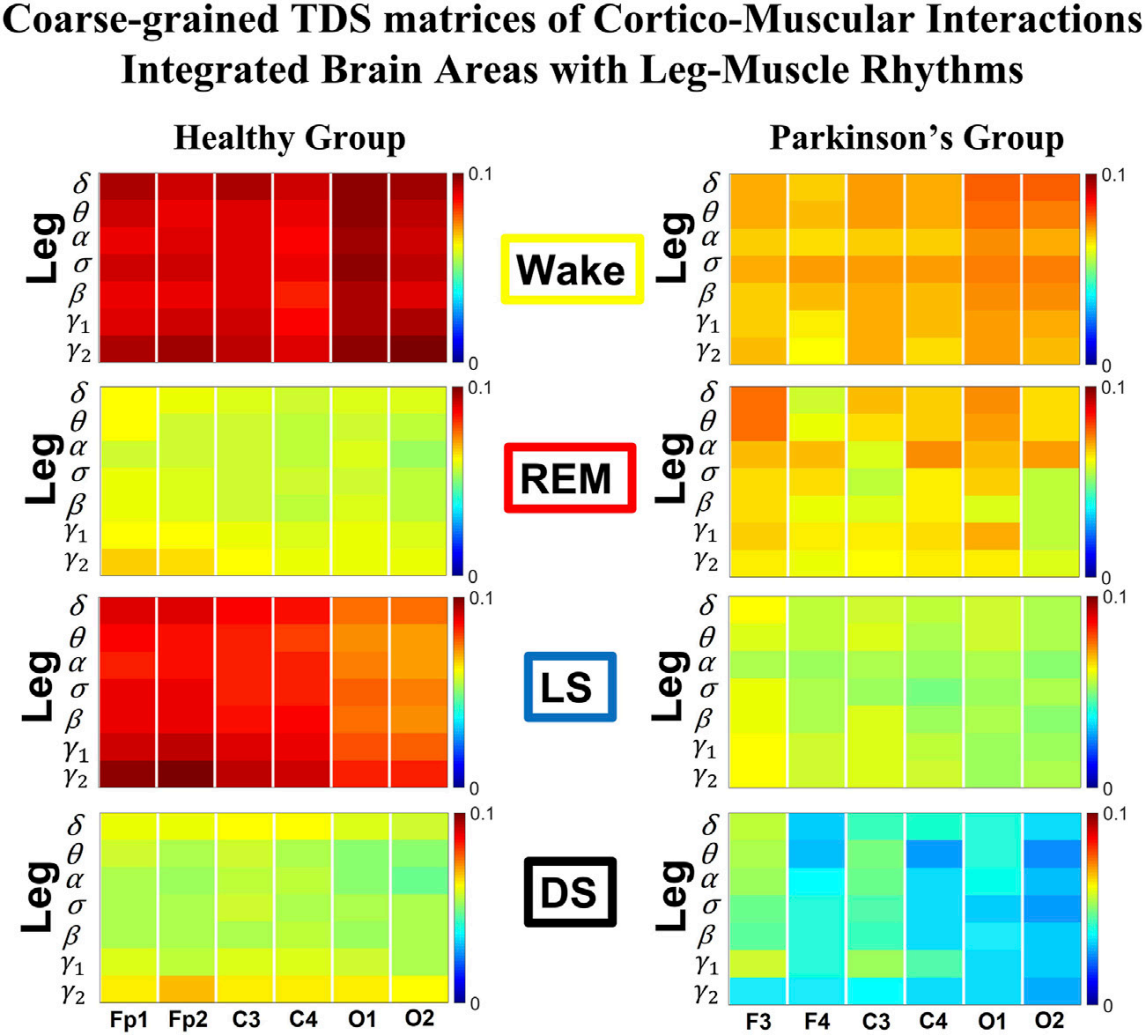
Dominant channels of communication and reorganization in leg-brain network interactions across physiological states in healthy and Parkinson’s subjects. Group-averaged matrices of coupling strength (measured as *%TDS*; see Methods 2.3) for brain vs. leg muscle tone interactions coarse-grained to represent the average coupling of each individual EMG frequency band with integrated spectral power of all cortical rhythms for different brain locations for healthy (left panels), and PD subjects (right panels). Brain-leg networks exhibit pronounced reorganization with transition across sleep stages for both physiologic and pathologic conditions. Physiologic brain-leg networks show stronger coupling during Wake and LS, and weaker coupling during REM and DS, while PD networks exhibit a gradual decline in link strength from Wake to REM, light and deep sleep. Moreover, for healthy, high frequency EMG rhythms (*γ*_1_ and *γ*_2_) exhibit stronger TDS coupling across all cortical areas (EEG channels) in healthy subjects (marked by warm colors); in PD subjects an opposite behavior is observed: strongest interactions during REM, LS and DS are generally mediated through low frequencies (*δ* and *θ*) across all cortical areas.

To understand the role of cortical areas in regulating distinct rhythms embedded in leg-muscle tone, we coarse-grain the TDS matrix for each state in Figure 1A, so that rows in each block of the coarse-grained matrix represent the average coupling of different leg-muscle rhythms (vertical axis) with a given cortical area represented by EEG channel location (horizontal axis) (Figure 11). In the following text these averaged coarse-grained matrices for each state are referred as muscle-to-brain interaction matrices for the healthy and PD group. The coarse-grained interaction matrices clearly exhibit a reorganization across physiologic states in both healthy and PD subjects. Healthy brain-leg networks show stronger coupling during Wake and LS, and weaker coupling during REM and DS. The Wake-REM-LS-DS alternation, very evident in healthy, breaks down with Parkinson’s, assuming a milder characterization and a change in the pattern. PD interaction matrices exhibit a gradual decline from Wake to DS. Moreover, we note that PD network interactions are weaker compared to healthy during Wake, LS and DS (colder colors in the matrices), and stronger during REM (warmer colors). In contrast to the coarse-grained brain-to-muscle interaction matrices (Figure 5), the leg-to-brain coarse-grained matrices in both healthy and PD subjects are characterized by homogeneous coupling strength for all leg-muscle tone rhythms and integrated cortical activity for the different cortical locations. We note that frontal and central cortical areas for healthy, and frontal cortical areas for PD are involved in the stronger interactions across all sleep stages.

**FIGURE 12:**
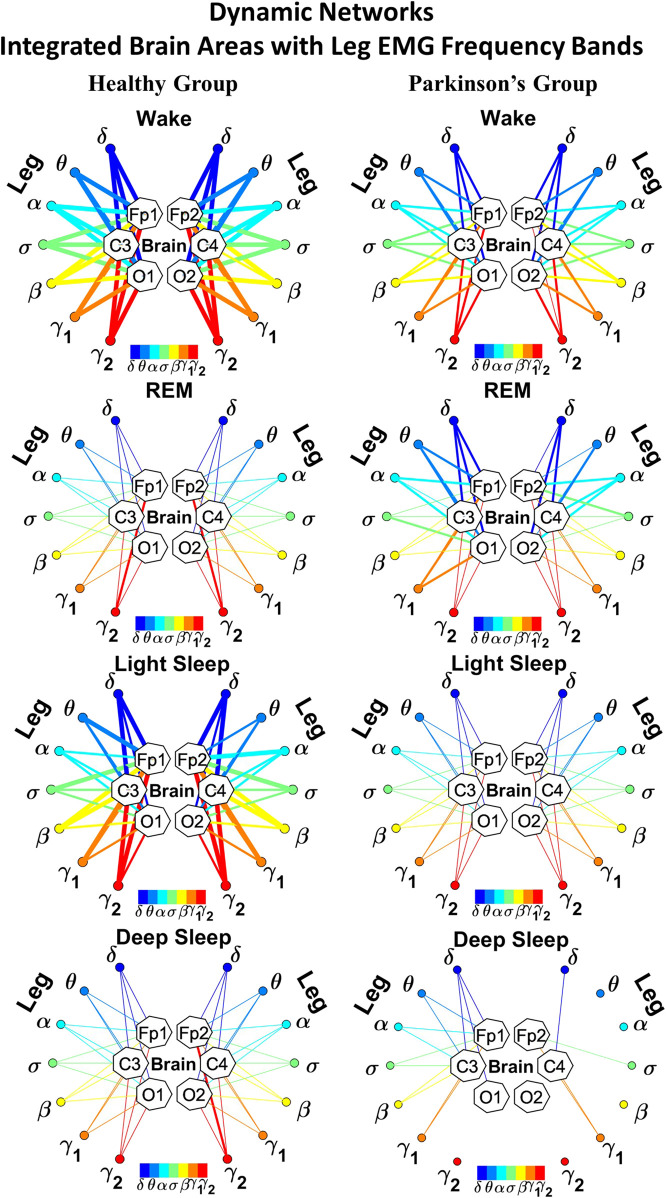
Dynamic networks of individual leg EMG frequency bands and integrated brain dynamics at cortical locations for different physiological states in healthy and Parkinson’s subjects. Links in network maps represent group-averaged TDS coupling strength (Section Methods 2.5.1) between each frequency band of leg muscle tone and a given cortical location, after averaging over all brain waves (see Section Methods 2.5.3) for healthy (left networks) and PD subjects (right networks), and correspond to the elements in the coarse-grained matrices shown in Figure 11. Brain areas are represented by Frontal (F3, F4), Central (C3, C4) and Occipital (O1, O2) EEG channels, while peripheral network nodes with different colors represent leg EMG frequency bands. Line thickness indicates link strength (thin links with 4% ≤ TDS <6.5% , intermediate links with 6.5% ≤ TDS <8.5% and thick links with TDS ≥8.5% ) and links color corresponds to the color of leg EMG frequency bands (network nodes). Network reorganization is observed with transition across sleep stages for both physiologic and pathologic conditions. Physiologic brain-leg networks show stronger coupling during Wake and LS, and weaker coupling during REM and DS, while PD networks exhibit a gradual decline in link strength from Wake to REM, light and deep sleep. The change in the sleep-stage pattern is due to a decrease of links strength for PD compared to healthy during Wake, LS and DS (sparser networks and thinner links) and an increase during REM (thicker links).

To visualize information present in the coarse-grained TDS matrices (Figure 11) we present our results in the corresponding leg-to-brain networks (Figure 12) for healthy (left networks) and PD subjects (right networks). PD interaction networks clearly show the change in the sleep-stage stratification pattern and an overall decrease in link strength compared to healthy (networks links thinner and almost absent in DS). We find that the coarse-grained leg-brain network is characterized by uniform distribution of links strength across cortical areas for both healthy and PD subjects, with exception of frontal areas in healthy and frontal and central cortical areas in PD, which are involved in the strongest interactions during REM. We note that during REM healthy networks show strong interactions between frontal brain areas and *γ*_2_-leg EMG frequency band (thick red links), while in PD subjects the strongest interactions during REM are engaged by low-frequency EMG bands *δ*, *θ* and *α* (thick dark and light blue links). Similarly to brain-to-leg interaction networks (Figure 6), the leg-to-brain networks exhibit a general symmetry between left and right hemispheres, with exception of PD during DS, where left frontal and central cortical areas are involved in stronger interactions compared to the other brain areas.

**FIGURE 13:**
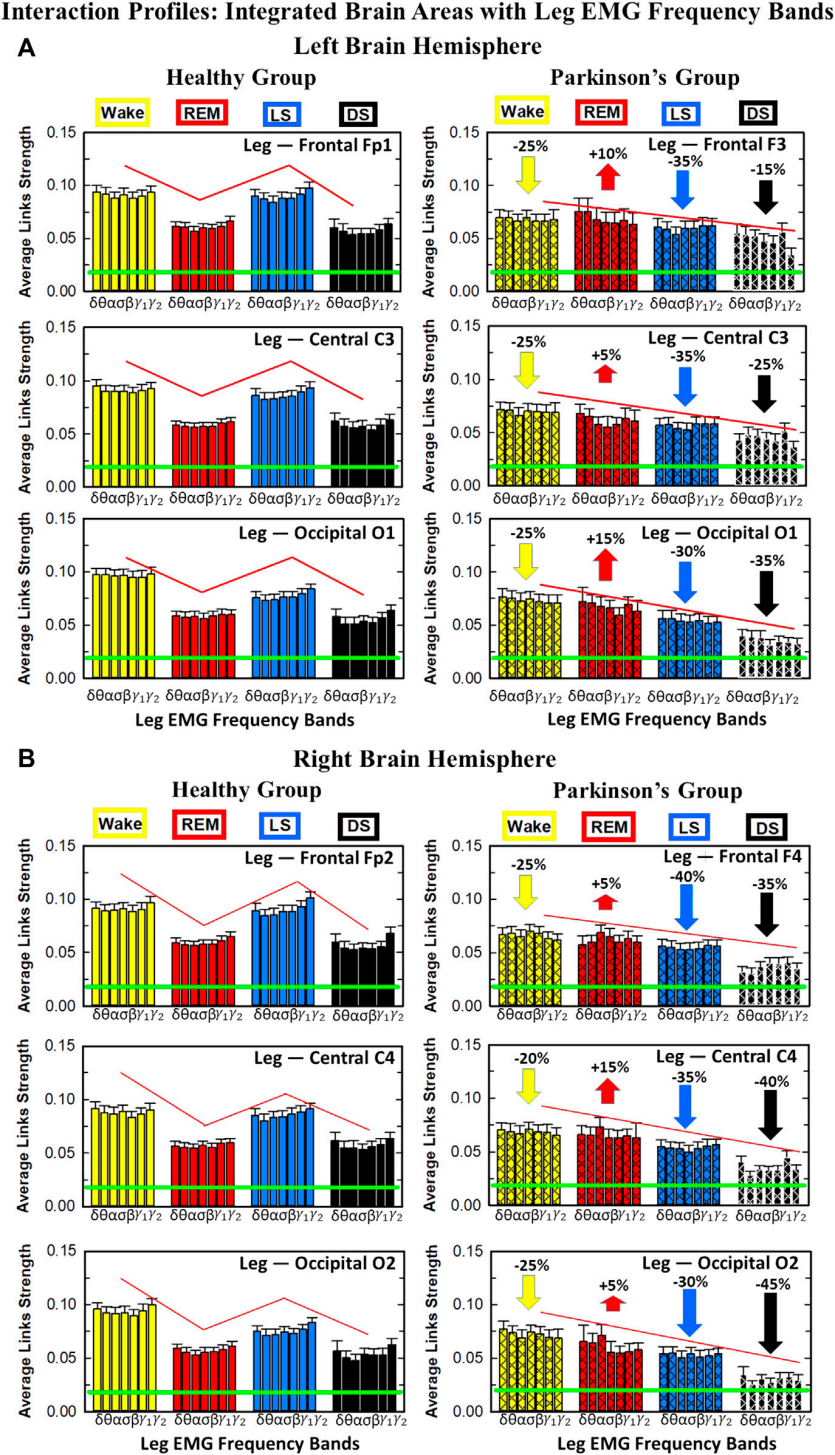
Characteristic profiles of network links strength representing interactions between integrated brain activity at cortical areas and individual leg-EMG frequency bands in healthy and Parkinson’s subjects. Group-averaged links strength are obtained using the TDS method (Methods Section 2.3), where each link represents the interaction of brain activity from a given cortical area in the left **(A)** or right brain hemisphere **(B)** (averaged over all brain waves derived from the EEG channel located at this cortical area) and each muscle tone rhythm (frequency band) derived from the leg-EMG signal in healthy (left panels) and PD subjects (right panels). Links are grouped by sleep stages and by brain cortical areas, and are ordered from low-to high-frequency leg-EMG bands. Bars indicate the strength of links shown on the network maps in Fig12. Note that the average strength of each group of links in the panels corresponds to a separate bar in Figure 6B. Error bars represent the standard error obtained for all subjects in each group; horizontal green lines mark the threshold *%TDS* = 2.3% (based on surrogate test, Section Method 2.4) for physiological significance. The reorganization of cortico-muscular networks with transitions across sleep stages is perturbed with PD. Brain-leg interactions in PD show a decrease of 15% − 35% in the left hemisphere **(A)** and 20% − 45% in the right hemisphere **(B)** during Wake, light and deep sleep (statistically significant difference – *p* ≤ 0.0272 Wilcoxon test for left and *p* ≤ 0.0299 Wilcoxon test for right hemisphere – during Wake and LS and borderline significant during DS) and an increase of 5% − 15% during REM in both hemispheres (*p* ≤ 10^−3^ Wilcoxon test for left, and *p* ≤ 0.0034 Wilcoxon test for right hemisphere). Bar-charts in both physiologic and pathologic conditions show absence of dominant links in the interactions of leg-EMG bands with cortical areas, and a flat profile of links strength across EMG bands for all cortical brain areas in both hemispheres during all sleep stages.

We found that PD affects the cortico-muscular networks and their reorganization across sleep stages in a complex way preferring certain brain waves as the main target in the brain-muscle communication. Therefore, we hypothesize that even the spectrum of different EMG frequency bands in PD networks will show an intricate mosaic of effects on the individual links. To this aim, we next study the characteristic profile of network links strength of interactions between individual leg rhythms and integrated cortical activity at a given cortical location in left (Figure 13A) and right brain hemispheres (Figure 13B) for healthy (left panels) and PD subjects (right panels). In contrast to the coarse-grained brain-leg networks (Figure 7), we do not observe dominant links in the interactions of individual leg EMG rhythms with integrated cortical activity at the different cortical areas of both left (Figure 7A) and right brain hemisphere (Figure 7B) for healthy subjects (left panels) during all sleep stages. Alternatively, PD interactions (right panels) show the slight dominance of low-frequency *δ* and *θ* leg-EMG bands for the left hemisphere in the cortico-muscular communication during REM, LS and DS; the frequency profile in the right hemisphere is similar, with the only difference that during REM *α* leg-EMG band engages the strongest interactions.

#### 3.3.2 Network interactions of integrated cortical activity with distinct rhythms in chin-muscle tone

**FIGURE 14:**
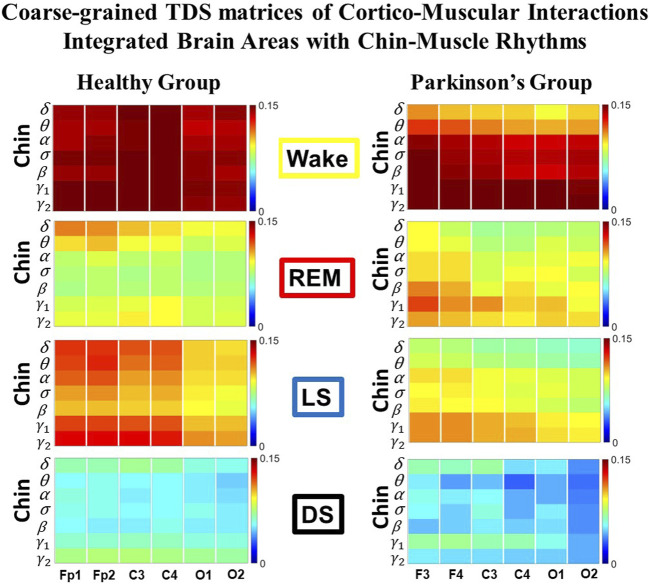
Dominant channels of communication and reorganization in chin-brain network interactions across physiological states in healthy and Parkinson’s subjects. Group-averaged matrices of coupling strength (measured as *%TDS*; see Methods 2.3) for brain vs. chin muscle tone interactions coarse-grained to represent the average coupling of each individual EMG frequency band with integrated spectral power of all cortical rhythms for different brain locations for healthy (left panels) and PD subjects (right panels). Brain-chin networks exhibit pronounced reorganization with transition across sleep stages for both physiologic and pathologic conditions. Physiologic brain-chin networks show stronger coupling during Wake and LS, and weaker coupling during REM and DS, while PD networks exhibit a gradual decline in link strength from Wake to REM, light and deep sleep. Note that in healthy subjects during REM low frequency *δ* and *θ* chin EMG bands exhibit stronger TDS coupling (marked by warm colors) compared to the other frequencies, while during LS the role of main mediator in the brain-chin interactions is shared with high frequency *γ*_1_ and *γ*_2_ chin EMG bands. Differently, in PD subjects high frequency *β*, *γ*_1_ and *γ*_2_ chin EMG bands are the main mediators in the brain-chin interactions across all sleep stages.

**FIGURE 15:**
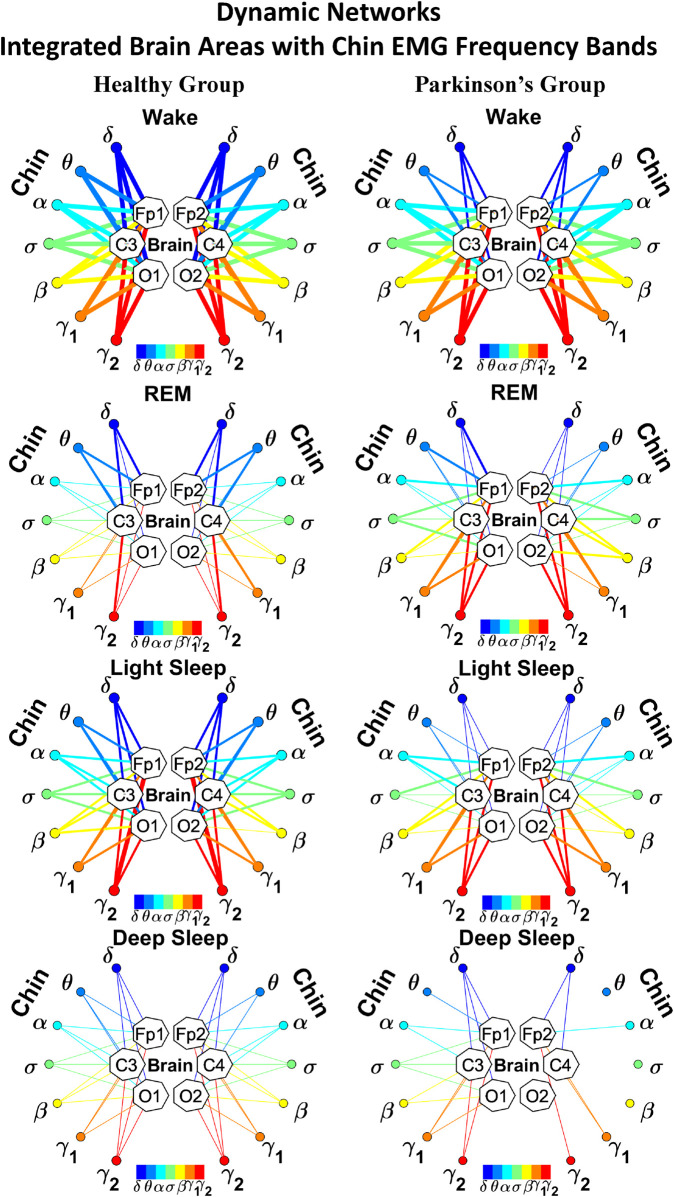
Dynamic networks of individual chin EMG frequency bands and integrated brain dynamics at cortical locations for different physiological states in healthy and Parkinson’s subjects. Links in network maps represent group-averaged TDS coupling strength (Section Methods 2.5.1) between each frequency band of chin muscle tone and a given cortical location, after averaging over all brain waves (see Section Methods 2.5.3) for healthy (left networks) and PD subjects (right networks), and correspond to the elements in the coarse-grained matrices shown in Figure 14. Brain areas are represented by Frontal (F3, F4), Central (C3, C4) and Occipital (O1, O2) EEG channels, while peripheral network nodes with different colors represent chin EMG frequency bands. Line thickness indicates link strength (thin links with 5% ≤ TDS <9% , intermediate links with 9% ≤ TDS <13% and thick links with TDS ≥13% ) and links color corresponds to the color of chin EMG frequency bands (network nodes). Network reorganization is observed with transition across sleep stages for both physiologic and pathologic conditions. Physiologic brain-chin networks show stronger coupling during Wake and LS, and weaker coupling during REM and DS, while PD networks exhibit a gradual decline in link strength from Wake to REM, light and deep sleep. The change in the sleep-stage pattern is due to a decrease of links strength for PD compared to healthy during LS and DS (sparser networks and thinner links).

We coarse-grain the TDS matrix for each state in Figure 3A to understand the role of cortical areas in regulating distinct rhythms embedded in chin-muscle tone. Thus, rows in each coarse-grained matrix block represent the average coupling of different chin-muscle rhythms (vertical axis) with a given cortical area represented by EEG channel location (horizontal axis) (Figure 14). The change in the sleep-stage stratification pattern with Parkinson’s is evident in the chin-to-brain matrices (Figure 14), as well as in the corresponding interaction networks (Figure 15). Furthermore, the PD brain-chin interactions are overall weaker compared to healthy across all sleep stages (sparser networks and thinner links). In both healthy and PD brain-chin interactions the most interested brain locations in the brain-muscle interactions are the frontal and central areas—Fp1/F3, Fp2/F4, C3, and C4 — which are closer to the motor cortex (warmer colors in the coarse-grained matrices and ticker links to these brain areas in the networks). The chin-to-brain networks (Figure 15) show a general symmetry between left and right hemisphere, with exception of PD during DS, where the left hemisphere is involved in stronger links than the right hemisphere, and a uniform distribution across different cortical areas, with exception for frontal and central EEG locations which are involved in the strongest interactions during REM and LS in healthy conditions. Specifically, during REM and LS the main mediators of the brain-muscle cross-talk are lowest frequencies *δ* and *θ* and highest frequencies *γ*_2_ chin EMG bands (thicker blue and red links). Differently from leg-to-brain interaction matrices, the dominant role of certain chin EMG frequency bands is observed for both healthy and PD subjects (Figure 14), characterizing the chin muscle group and differentiating it from the leg muscle group. In healthy subjects during REM low-frequency *δ* and *θ* chin EMG bands exhibit stronger TDS coupling (marked by warm colors) compared to the other frequencies, while during LS the role of main mediator in the brain-chin interactions is shared with high-frequency *γ*_1_ and *γ*_2_ chin EMG bands. Differently, in PD subjects high frequency *β*, *γ*_1_ and *γ*_2_ chin EMG bands are the main mediators in the brain-chin interactions across all sleep stages. This is a demonstration that a change in the network structure not only characterizes specific physiologic states, but also allows us to discriminate between physiologic and pathologic conditions.

**FIGURE 16:**
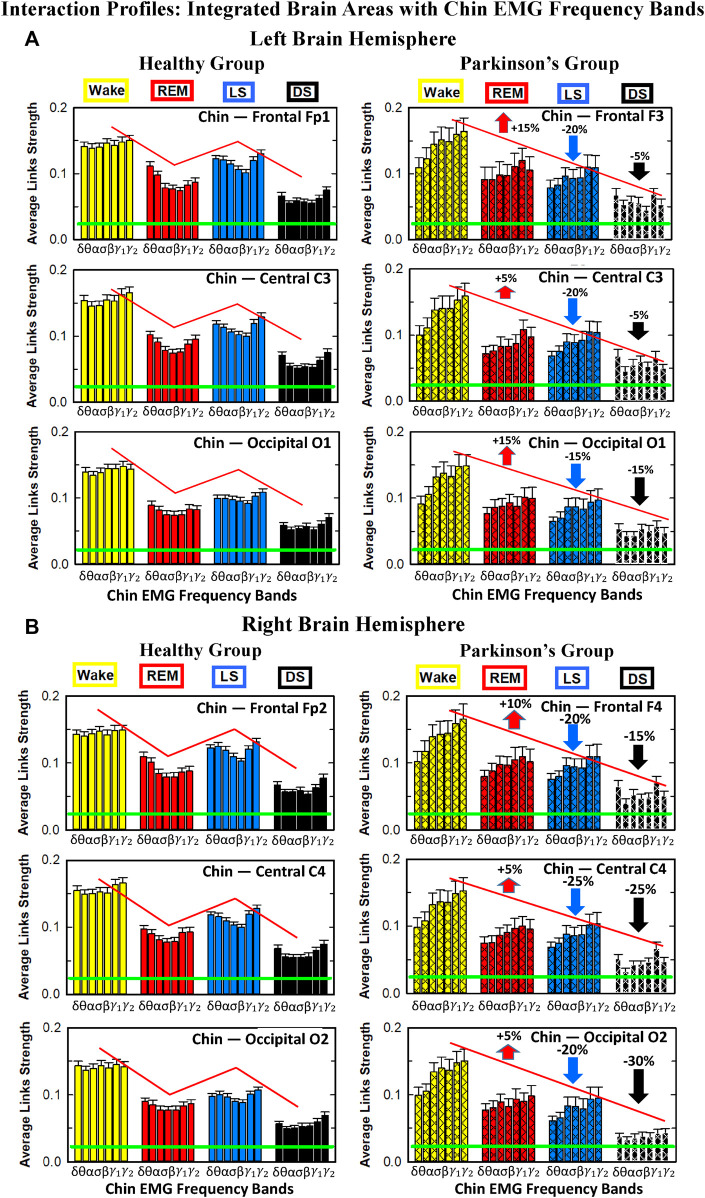
Characteristic profiles of network links strength representing interactions between integrated brain activity at cortical areas and individual chin-EMG frequency bands in healthy and Parkinson’s subjects. Group-averaged links strength is obtained using the TDS method (Methods Section 2.3), where each link represents the interaction of brain activity from a given cortical area in the left **(A)** or right **(B)** brain hemisphere (averaged over all brain waves derived from the EEG channel located at this cortical area) and each muscle tone rhythm (frequency band) derived from the chin-EMG signal in healthy (left panels) and PD subjects (right panels). Links are grouped by sleep stage and by brain cortical areas, and are ordered from low-to high-frequency chin-EMG bands. Bars indicate the strength of links shown on the network maps in Figure 15. Note that the average strength of each group of links in the panels corresponds to a separate bar in Figure 9B. Error bars represent the standard error obtained for all subjects in each group; horizontal green lines mark the threshold *%TDS* = 2.3% (based on surrogate test, Section Method 2.4) for physiological significance. The reorganization of cortico-muscular networks with transitions across sleep stages is perturbed with PD. Brain-chin interactions in PD show a decrease of 5% − 20% in the left hemisphere **(A)** and 15% − 30% in the right hemisphere **(B)** during light and deep sleep (statistically significant difference – *p* ≤ 0.0260 Wilcoxon test for left and *p* ≤ 0.0164 Wilcoxon test for right hemisphere–during LS, with exception of O1 – *p* = 0.0939 Wilcoxon test – and borderline significant during DS) and an increase of 5% − 15% and 5% − 10% during REM for left and right hemisphere respectively (*p* ≤ 0.0208 Wilcoxon test for the left and *p* ≤0.0089 Wilcoxon test for the right hemisphere). A characteristic profile of network links strength as function of chin EMG frequency bands is consistently observed for all brain areas during REM, light and deep sleep for healthy subjects. The frequency profile as function of chin EMG frequency bands changes in PD subjects: the low frequency cortical rhythms *δ* and *θ* are in general the most affected and a clear decrease in link strength is observed in Wake, REM and LS.

We discovered that for healthy subjects the interactions of individual chin-muscle rhythms with integrated cortical activity at different cortical areas are characterized by a set of distinct profiles for network links strength that is universal for all subjects during each sleep stage (Figure 16). Further, we find that in healthy subjects with transition from one sleep stage to another these interaction profiles change—e.g., wake is characterized by a homogeneous interaction profile with a uniform distribution of links strength for all chin-muscle rhythms; during REM the interaction profile exhibits stronger links involving *δ* and *θ* chin-EMG rhythms, while during LS and DS both low-frequency *δ* and *θ* as well as high-frequency *γ*_1_ and *γ*_2_ are main mediators of the interaction (left panels in Figures 16A,B). These profiles specific for a given physiologic state dramatically change with Parkinson’s, where a profile with high-frequency as main mediators, followed by medium and then low-frequency EMG bands–*γ*_2_, *γ*_1_, *β*, *σ*, *α*, *θ* and *δ*–is consistently observed across all cortical areas and almost all sleep stage, with the only exception of DS, which presents a more uniform distribution. This characteristic let us conclude that, differently from the brain-to-muscle interactions, where PD affects more the high-frequency cortical rhythms, in the muscle-to-brain interactions the low-frequency EMG bands are the main target of PD. Again, the whole spectrum of different cortico-muscular interactions involving different frequency bands allows us to distinguish between one physiologic state to another, and between healthy and pathologic conditions.

The uncovered links strength profiles for the distinct modules in the cortico-muscular network represent short-time scales synchronous modulation in the bursting activity of cortical and muscle rhythms embedded in the spectral power of EEG and EMG dynamics. These profiles indicate a hierarchical organization in the entire cortico-muscular communication network that is specific for each physiological state. With transition across states the entire cortico-muscular network reorganizes leading to change in the links strength profile in all network modules. Further, this network structure and profiles of links strength dramatically change with neurodegenerative disorders, such as Parkinson’s, leading to different pattern of interaction and different role of cortical and muscular rhythms in cortical-muscular regulation. The observed differences are consistent for two different muscle groups (leg and chin) and are universal across PD subjects, opening a new class of biomarkers for early diagnosis and prognosis of Parkinson’s.

## 4 Discussion

The general paradigm in research on functional connectivity between the brain and the locomotor system in health as well as in motor and neurodegenerative disorders such as Parkinson’s has primarily focused on the association between specific movements and responses in brain activity. Empirical investigations in PD have reported elevated amplitude and spectral power for certain cortical rhythms, in particular *β*-waves, with current drug treatments and deep brain stimulation procedures aiming to reduce *β*-power (Benabid, 2003). Recent works have uncovered certain types of coupling (phase-amplitude, phase-synchronization) for specific pairs of cortical rhythms (e.g., *β* − *γ*) and coherence between EEG and EMG dynamics in specific narrow frequency bands, however, always in the context of particular movement tasks or exercises (walking, running, *etc.*) (Conway et al., 1995; Boonstra et al., 2009; Chen et al., 2013b; Cheyne, 2013; Muthuraman et al., 2018). There is not comprehensive understanding of cortico-muscular connectivity involving all cortical rhythms across different brain locations and multiple EMG rhythms embedded in different type muscles. Here we study functional brain-muscles interaction networks in healthy and PD subjects considering the entire set of physiologically relevant brain waves and EMG rhythms, and we focus on basi physiological states such as quiet wake, sleep and sleep stages, when the muscle activity is low and neuro-autonomic control is dominant. We establish how these networks reorganize with transitions across physiological states in response to change in autonomic regulation and in the absence of targeted movements. Specifically, we consider the network interactions between seven EEG cortical rhythms and seven EMG rhythms in leg and chin muscle tone during Wake, LS, REM, and DS, and we uncover basic functional pathways of cortico-muscular cross-communication that characterize each physiologic state in both healthy and pathologic conditions. Our empirical findings indicate that future treatment strategies should restore not just the characteristics and dynamics of individual cortical rhythms in relation to physiological states and clinical conditions (Benabid, 2003; De Hemptinne et al., 2015), but also to address the network of interactions between cortical rhythms and rhythms of muscle activity.

Understanding the mechanism cortico-muscular control during sleep is important, not only to uncover default networks of brain-muscle cross-communication, and how these networks facilitate transitions from one physiological state to another, but also to identify correlates between Parkinson’s and sleep behavior disorders that manifest in the early stages of the disease (Chaudhuri et al., 2002; McKeith et al., 2005; Cochen De Cock and Arnulf, 2008). It is known that subjects with PD are first claimed to exhibit motor dysfunction related to REM behavior disorder years before standard diagnosis related to tremor and freezing of gait (Gagnon et al., 2002; Iranzo et al., 2006; Postuma et al., 2009). Therefore, the established here network underlying brain control of the muscle activity during sleep in healthy and PD conditions sheds light on the connection between sleep behavior disorder and PD, with implications for early diagnosis.

### 4.1 Reorganization of cortico-muscular interaction networks across sleep stages for healthy and PD subjects

Recent findings in the new field of Network Physiology demonstrate the unique correspondence between systems interactions and physiological state (Bartsch et al., 2012; Bartsch et al., 2014; Bartsch and Ivanov, 2014; Borovkova et al., 2022; Clemson et al., 2022; Froese et al., 2022; Schöll et al., 2022). Similarly, we find that cortico-muscular interaction networks reorganize their structure and topology across sleep stages for both leg and chin muscle tone activation, and this reorganization changes with PD (Figure 1–Figure 4), leading to different network structures as a result of pathological regulation. While healthy networks interactions show an up-down-up-down stratification pattern across sleep stages, with stronger links during Wake and LS and weaker during REM and DS, PD networks exhibit a gradual decline from Wake to DS (Figure 6; Figure 9, Figure 12; Figure 15). This change is due to a decline in links strength during Wake, LS and DS, and an increase during REM. It has been found that PD patients frequently exhibit RBD, showing an increased muscle activity during REM (Gagnon et al., 2002; Iranzo et al., 2006; Postuma et al., 2009). Our findings demonstrate the presence of a new fundamental characteristic of RBD related to the synchronous activation of cortical rhythms and rhythms of muscle activity. Although RBD is associated to low EMG amplitude for muscular activity, our method is independent of the amplitude of the EMG signal, and can still identify and quantify synchronous modulations in the EEG and EMG signals. In fact, signals with small amplitude, or one dominant signal with large amplitude and another one with small amplitude, can have a strong coupling expressed in synchronous modulations (bursts) in their respective dynamics, that can be detected by our TDS method. Therefore, EMG activity reflected in the amplitude of the EMG signal and presence of interactions between two systems are two distinct concepts. Our results are in line with previous findings on RBD in PD patients, but also demonstrate a new aspect of RBD: the network connectivity and network link strength between cortical and muscular rhythms increases during REM.

### 4.2 Muscle-specific effects of Parkinson’s on cortico-muscular networks

While traditionally empirical investigations of PD have focused on responses in brain dynamics to locomotor tasks and targeted movements, here we study complex networks of cortico-muscular interactions representing synchronization among brain waves and muscle rhythms embedded in different muscles, and we track the evolution of these networks across sleep stages, comparing healthy and PD subjects. We demonstrate that neurodegeneration due to PD significantly affects neuro-autonomic control of locomotion during sleep, when physical activity level is greatly reduced. The uncovered reorganization in the sleep-stage stratification pattern for the cortico-muscular networks in PD subjects, characterized by gradual decline in connectivity and links strength from wake to DS, reveals loss of flexibility in brain-muscle communication networks with transition across states. Consequently, the autonomic mechanism of locomotor control loses adaptability to changes in physiologic states, that in turn may lead to a general disorder in physiologic system interactions across the entire organism. Further, cortico-muscular network connectivity in PD significantly decreases during LS and DS–an important observation, since there are no prior reports of PD neurodegeneration affecting these two states. This demonstrates that the uncovered here increase in cortico-muscular links strength during REM, that parallels increase in muscle activity associated with REM behavior disorder, as well as the decline in link strength during LS and DS, can serve as novel markers for early-phase diagnosis of PD disorder. Moreover, we note that both brain-chin and brain-leg interaction networks reorganize with transition across sleep stages, however, with much stronger effect of PD disorder on the brain-leg network. This indicates that the cortico-muscular network responses to PD degeneration may be muscle specific, thus opening new areas of investigation. Our empirical study uncovers basic laws of autonomic control on muscle activity in the absence of locomotor tasks and targeted movements, thus, providing a link between PD disorder and prodromal symptoms (absence of muscle atonia during REM, fragmented sleep) for early diagnosis based on novel network-derived biomarkers.

### 4.3 Unique correspondence between physiological state and brain-muscle network interactions profile

Our findings demonstrate that each physiological state is uniquely defined by a particular profile of interactions among cortical EEG and muscle EMG rhythms and how this profile changes with PD. We distinguish two muscles: chin and leg. In the brain-leg interaction network, cortical rhythms *γ*_1_ and *γ*_2_ are the dominant channels of cortico-muscular communication during quiet wake and sleep stages in healthy conditions. In contrast, for the PD group *γ*_1_ and *γ*_2_ cortical rhythms share dominant role with the *δ* cortical rhythm that is consistently observed across cortical locations and sleep stages (Figure 7). Such change in the network links strength interaction profile shows that the effect of PD is not only “global”, homogeneously altering the overall strength of network links for each sleep stage, but there is also a ‘local’ effect altering more significantly the coupling of certain cortical rhythms with muscle activity. For the brain-chin network in healthy subjects, the links strength interaction profile across different brain waves is similar to the healthy brain-leg interactions profile. In contrast, the interaction profile of the brain-chin network in PD subjects is not affected and remains similar to healthy subjects for all cortical locations and sleep stages, exhibiting only a ‘global’ PD effect on the overall brain-chin links strength (Figure 10).

Investigating the individual role of EMG rhythms in cortico-muscular communication with integrated cortical areas, we note that also in this case the brain-leg and brain-chin networks follow dynamics characterized by different links strength interaction profiles. In the brain-leg network the interactions profiles for healthy subjects are generally flat for all cortical areas and sleep stages, showing absence of dominant muscle rhythms, while in contrast interaction profiles for PD subjects are characterized by dominant role of low frequency *δ* and *θ* EMG rhtyhms (Figure 13). On the other hand, brain-chin interactions exhibit a different behavior compared to brain-leg interactions: i) under healthy conditions there are no dominant EMG rhythms in the network communication during Wake, while low frequency muscle rhythms are pronounced during REM, LS and DS (Figure 16 left panels); ii) in PD subjects the brain-chin interaction network exhibits a different profile, where high frequency muscle rhythms play dominant role in the brain-muscle communication across all cortical areas and sleep stages (Figure 16 right panels). Further, we note that PD affects differently the rhythms in distinct muscles: i) for brain-leg interaction networks a decrease in the coupling between high frequency muscle rhythms and integrated cortical activity is paralleled by increased coupling of low frequency muscle rhythms with cortical activity; ii) in contrast, brain-chin interactions exhibit increase in the coupling between high frequency muscle rhythms and integrated cortical activity, that is paralleled by decreased coupling of low frequency muscle rhythms and cortical activity. These differences in the cortico-muscular networks and interaction profiles for the leg and chin muscles reflects the role of different muscle histochemical composition, with different control through motor neurons and differentiated impact of Parkinson’s neurodegeneration on network structure, and is consistent with previous studies showing frequency-specific responses of muscle activity to exercises and fatigue-related stress (Garcia-Retortillo et al., 2020; Garcia-Retortillo and Ivanov, 2022). Such muscle-specific differentiation in cortico-muscular interactions can provide new insights on locomotor control under autonomic regulation and new diagnostic tools to assess effects of REM behavior disorder.

### 4.4 Novelty

Dynamics of EEG signals and brain waves during sleep have been extensively studied in the literature in the context of healthy sleep, sleep disorders and Parkinson’s. Elevated *β*-wave power (Benabid, 2003) and increased *θ* and *β* synchronization across the cortex has been reported in Parkinson’s (Stoffers et al., 2008; Krause et al., 2014). Further, brain dynamics, brain-brain interaction networks and functional forms of coupling between brain waves have been investigated in the context of sleep (Liu K. K. et al., 2015; Lin et al., 2020). Here we analyze the coupling between brain waves and muscle rhythms, and we establish the underlying functional networks and their reorganization in response to changes in autonomic regulation with transition across sleep stages, thus, uncovering previously unknown aspects of cortico-muscular control. Moreover, we note that modulation (elevation or suppression) in EEG/EMG spectral power previously reported in Parkinson’s does not affect the degree of coupling between cortical and muscular rhythms as quantifies by the TDS method (Methods Section 2.3) and does not affect the results of our network analyses–the TDS method quantifies the degree of synchronization between bursts in brain waves and muscle rhythms and does not depend on the amplitude of the EEG and EMG signals. In fact, a pair of signals with small amplitude, or a pair where one signal is dominant with large amplitude and the other has small amplitude, can exhibit strong coupling as quantified by synchronous bursts in their respective dynamics. Thus, our study provides new independent information about cortico-muscular control and its modulation with neurodegeneration.

### 4.5 Study limitations

We note that in our study we did not differentiate between subjects with different degree of PD severity (Hoehn *&* Yahr stages). Stratifying results across different PD stages, would have important clinical implications. However, the sample size of subgroups in our database corresponding to severity stages does not allow statistically meaningful analyses with low confidence intervals (our PD group consists of only 33 subjects). This is a limitation of our study. The primary aim of this pilot study is to identify and quantify the networks underlying cortico-muscular interactions, and how network dynamics and organization are affected by changes in autonomic regulation during wake and sleep stages, and by neurodegenration due to PD. In a follow-up study, based on much larger PD population, with statistically large subgroups of subjects with different Hoehn *&* Yahr stages, we plan to stratify the cortico-muscular interaction networks and investigate their characteristics as a function of disease severity. This would lead to potentially more precise network-based biomarkers tailored to comprehensively assess patients’ disease progression, response to medications and treatment.

### 4.6 Summary and clinical implications

Basic and clinical investigations of cortico-muscular control in healthy subjects and Parkinson’s disease have primarily focused on the association between specific movement tasks and corresponding responses of brain dynamics under various clinical conditions, medications and treatment strategies. A limited number of studies have analyzed direct forms of coupling between EEG and EMG signals, all in the context of walking and targeted movements. This work presents a first pilot study of direct cortico-muscular interactions during overnight sleep in the absence of conscious targeted movements and when autonomic regulation is dominant. Our findings demonstrate presence of dynamic network interactions with complex hierarchical organization that underlie cortico-muscular control. These networks involve all physiologically-relevant brain waves across cortical areas and activity rhythms embedded in different muscles. Further, we uncover that the cortico-muscular functional networks reorganize with sleep-wake and sleep-stage transitions, and breakdown with Parkinson’s neurodegeneration. Specifically, we find that network connectivity and links strength exhibit unique sleep-stage stratification under healthy condition that is significantly altered with PD. Moreover, we find that each physiological state (wake/sleep stages) is characterized by a specific profile of network links strength.

In the context of basic physiology the presented here Network Physiology approach and reported findings provide new insights on the laws of organ systems cross-communication and the principles of integration to facilitate various functions during distinct physiological states. The empirical analyses show a complex coordination and network integration among cortical rhythms and rhythms in muscle activity that respond to changes in autonomic regulation, with potential for novel network-based markers for early diagnosis, quantifying effects of medications and for guiding treatment strategies. In the context of sleep, we find that Parkinson’s neurodegenration perturbs cortico-muscular control during light and deep sleep (breakdown in network connectivity and decline in interactions strength), and that, in addition to elevated muscle tone, REM behavior disorder in PD is characterized by reinforced cortico-muscular network interactions.

## Data Availability

Data used in this work are pre-existing de-identified multi-channel EEG and EMG recordings and sleep-stage annotations for healthy subjects from the EU SIESTA database, and for Parkinson’s subjects from the Sleep Disorders Center database at Boston Medical Center. The detailed protocol of the SIESTA database can be found in Klösch et al. (2001). Data can be obtained upon request through the advisory board of the SIESTA Group (www.thesiestagroup.com), and Boston Medical Center.
